# Single Cerebral Organoid Mass Spectrometry of Cell-Specific Protein and Glycosphingolipid Traits

**DOI:** 10.1021/acs.analchem.2c00981

**Published:** 2023-02-01

**Authors:** Markéta Nezvedová, Durga Jha, Tereza Váňová, Darshak Gadara, Hana Klímová, Jan Raška, Lukáš Opálka, Dáša Bohačiaková, Zdeněk Spáčil

**Affiliations:** † RECETOX, Faculty of Science, Masaryk University, Brno 625 00, Czech Republic; ‡ Department of Histology and Embryology, Faculty of Medicine, Masaryk University, Brno 625 00, Czech Republic; § International Clinical Research Center (ICRC), St. Anne’s University Hospital, Brno 656 91, Czech Republic; ∥ Department of Chemistry, Faculty of Pharmacy, Charles University, Hradec Králové 500 05, Czech Republic

## Abstract

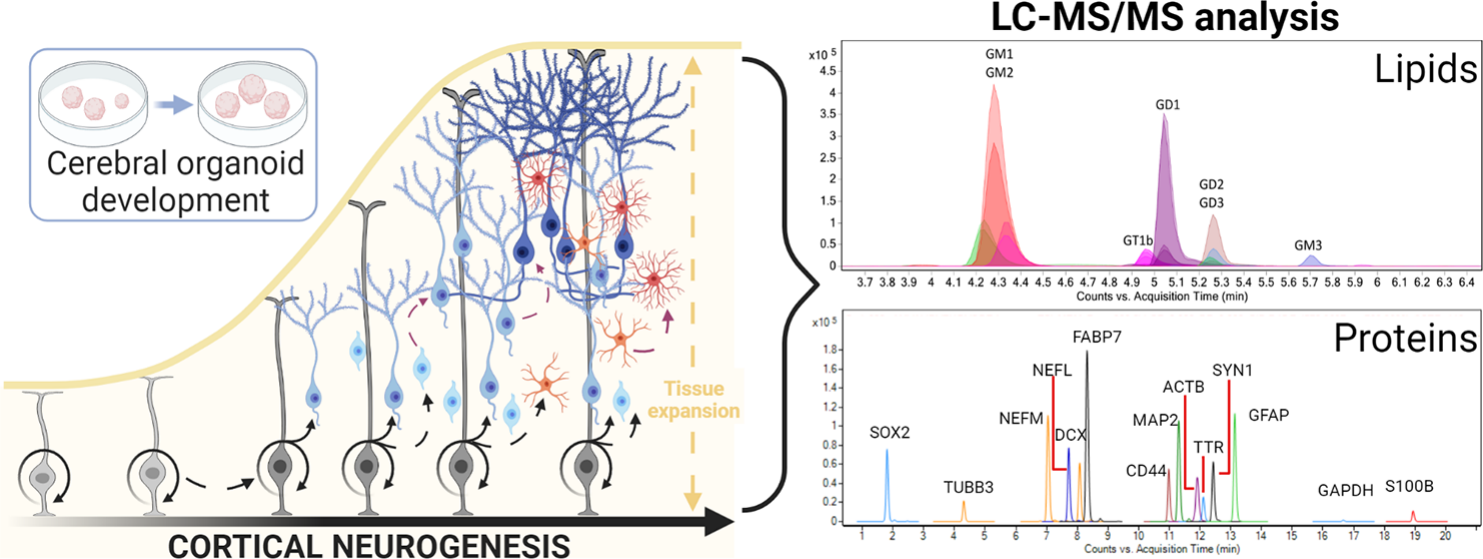

Cerebral organoids are a prolific research topic and an emerging model system for neurological diseases in human neurobiology. However, the batch-to-batch reproducibility of current cultivation protocols is challenging and thus requires a high-throughput methodology to comprehensively characterize cerebral organoid cytoarchitecture and neural development. We report a mass spectrometry-based protocol to quantify neural tissue cell markers, cell surface lipids, and housekeeping proteins in a single organoid. Profiled traits probe the development of neural stem cells, radial glial cells, neurons, and astrocytes. We assessed the cell population heterogeneity in individually profiled organoids in the early and late neurogenesis stages. Here, we present a unifying view of cell-type specificity of profiled protein and lipid traits in neural tissue. Our workflow characterizes the cytoarchitecture, differentiation stage, and batch cultivation variation on an individual cerebral organoid level.

Cerebral organoids (COs) generated from induced pluripotent stem cells (iPSCs) are an emerging in vitro model system in neurobiology.^1^ COs recapitulate human brain cytoarchitecture and cell diversity during neurogenesis, mimicking brain development in three dimensions.^2^ COs are increasingly used to model diseases-in-the-dish with recent viral applications toward Zika virus^3^ or SARS-COV-2.^4^ However, current cultivation protocols are notorious for substantial intra- and interbatch variation in differentiation, morphology, and cell composition.^5^

CO-based disease models expanded our ability to study neurodevelopment and degeneration via cell lineage-specific protein and lipid markers (Table S1 and Figure S1). At the early cortical neurogenesis stage, neural stem cells (NSCs) differentiate into radial glial cells (RGCs), giving rise to neurons, astrocytes, and oligodendrocytes.^5^ RGCs divide asymmetrically to generate neurons directly or indirectly through intermediate progenitor cells (IPCs), later differentiating symmetrically into immature neurons.^5^ NSCs express the early neurogenesis marker, a transcription factor SOX2. SOX2 is downregulated in post-mitotic neurons. Glial hallmarks (fatty acid-binding protein, FABP7) begin to emerge during later differentiation simultaneously with primary astrocyte markers—calcium-binding protein B (S100B), glial fibrillary acidic protein (GFAP), and CD44 antigen.^6^ Astrocytes express S100B during the proliferative and migration phase.^7^ A microtubule-associated protein 2 (MAP2) in neurons’ dendrites and reactive astrocytes stabilizes the microtubules against depolymerization.^8^ Tubulin beta-3 chain (TUBB3), the principal constituent of microtubules in neuronal axons, and microtubule-associated protein doublecortin (DCX) are characteristic of the immature neuronal population.^9^ DCX ceases with neuronal maturation.^9^ Mature neurons express neurofilaments containing intermediate filament proteins (light—NEFL, medium—NEFM) and synapsin-1 (SYN1). The choroid plexus’s epithelial cells representing the non-neuronal cells express the transthyretin (TTR).^9−11^

Like proteins, lipids constitute the primary structural aspect of neuronal membranes. Major cerebral lipids consist of phospholipids, glycolipids, cholesterol, and triglycerides. However, our work focused on membrane glycosphingolipids, particularly gangliosides, in examining primary neural development and maturation traits as they parallel protein cell-specific markers. Gangliosides are ubiquitous in vertebrate tissues and highly abundant in neural cells, essential for cellular signal transduction, adhesion, proliferation and differentiation, immune response, and apoptosis.^12^ Neuronal membranes and myelin sheaths contain 10–12% of gangliosides arranged in microdomains, referred to as lipid rafts.^13^ The perturbed composition of neuronal gangliosides in the membrane triggers neurodegeneration.^14^ The gangliosides’ distribution is associated with specific cell types and characterizes the cortical neurogenesis stage and cytoarchitecture in COs.

Cell-specific protein markers are frequently profiled in COs using antibody-based immunoaffinity assays, i.e., ELISA, Western Blot, or immunofluorescence staining.^15^ However, the quantitative performance, robustness, multiplexing capacity, and throughput of immunoaffinity assays are limited.^16^ Similarly, the thin-layer chromatography (TLC) immunostaining, liquid or gas chromatography (LC/GC) methodology to probe lipid composition often lacks sensitivity and selectivity.^17^ Few studies utilized organoid sections for immunostaining but struggled with the lack of diversity in information regarding the lipid subclasses.^18,19^ Organoids were often pooled before TLC analysis, hiding the level of heterogeneity.^20^ On the contrary, mass spectrometry (MS) proteomics^21^ and lipid profiling^22^ via selected reaction monitoring (SRM) assays are highly reproducible and quantitative.

We present a workflow to simultaneously profile cell-specific protein markers and glycosphingolipids in a single cerebral organoid to characterize cytoarchitecture and to identify outliers and the batch-to-batch variation^5^ (Figure 1). We used the bottom-up SRM protein assays, selecting surrogate proteotypic peptides to generate an SRM library. As the consensus on proteotypic peptide selection is missing, we report on the design of SRM protein assays (Figure S2).

**Figure 1:**
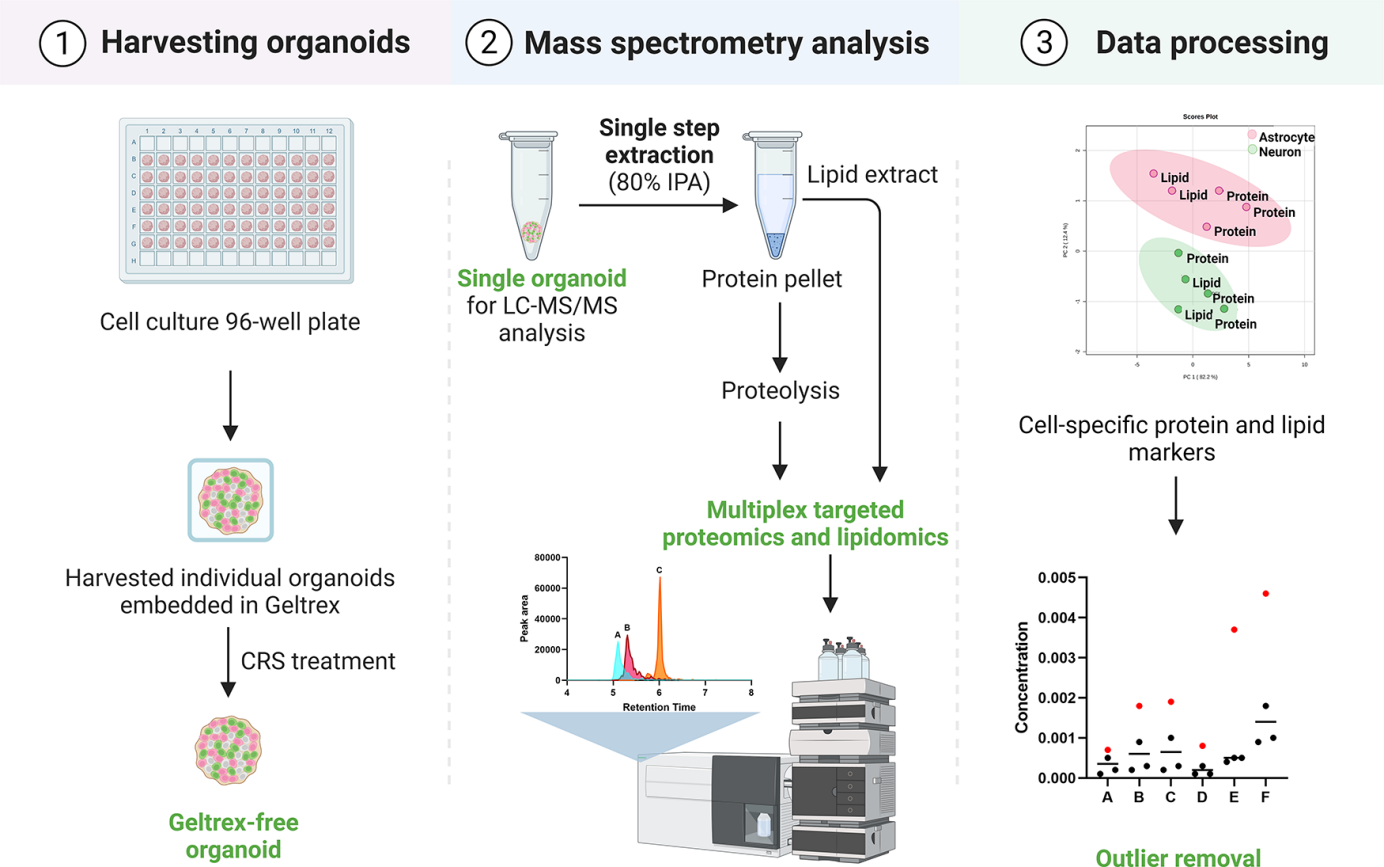
Single cerebral organoid mass spectrometry-based protein and lipid profiling. The workflow overview. Cerebral organoids were harvested after 48, 76, 95, 110, 135, and 160 days of differentiation, treated with the cell recovery solution to remove the cell culture matrix, and lipids were extracted using 80% IPA, and the protein pellet was subjected to the bottom-up SRM protein assays. Single-organoid protein and lipid profiling was the basis for cell-specific population characterization and the outlier removal to mitigate the intra- and interbatch variability.

## Experimental Section

### Lipid Extraction for Mass Spectrometry Assays

COs harvested for SRM analysis were immediately washed with PBS, treated (4 °C; 1 h) with cell recovery solution (CRS, Corning, New York), and washed again. COs were freeze-dried (γ 1–16 LSCplus, Martin Christ GmBH, Germany) and stored at −80 °C until further processing. For lipid and protein analysis, a single CO was used; biological replicates (*n* = 4) per time point were analyzed in duplicates. Freeze-dried CO was homogenized by adding 100 μL of water in a Protein LoBind (Eppendorf, Germany) microtube with a glass bead (Benchmark Scientific, Edison, New Jersey), sonicated, and vortexed. The homogenate was centrifuged briefly, and 10 μL of the supernatant was used to determine the total protein content by the BCA assay. The remaining homogenate was dried (Savant SDP121 P, SpeedVac, Thermo Fisher Scientific). For lipid extraction, we added 100 μL of 80% IPA to the dry homogenate, vortexed (1 min), sonicated (37 Hz, 5 min), and mixed (10 min, 2000 rpm). The sample was centrifuged (12.3 RCF for 5 min), and 85 μL of the lipid extract was removed from the residual protein pellet. Lipid extracts were stored at −20 °C until analysis. After lipid extraction, protein pellets were dried (SpeedVac, 37 °C) and processed for SRM protein assays (Figure 1).

### Mass Spectrometry Ganglioside Assays and Data Processing

Lipid extracts were twofold diluted by adding 0.3 μM of isotopically labeled GM1 and GM3 in 10% isopropanol (IPA). Sample volume (2 μL) was injected in a UHPLC system (1290 Infinity II; Agilent Technologies, California) equipped with C18 precolumn and analytical column (CSHTM, 5 × 2.1 mm^2^ × 1.7 μm and 50 × 2.1 mm^2^ × 1.7 μm from Waters Corp) thermostated at 40 °C. UHPLC system was coupled to a triple quadrupole mass spectrometer (Agilent 6495B, Agilent Technologies).

The mobile phase for the positive ion mode analysis consisted of buffer A (0.5 mM ammonium fluoride in the water) and B (methanol: IPA (50:50 v/v)). The gradient elution (17.1 min) at a flow rate of 0.3 mL/min was 30% B for 2 min, 70% B from 2 to 9 min, 95% B maintained from 9 to 13.3 min, 5% B at 13.3 min, and 5% B at 14.3 min with re-equilibration from 14.5 to 17.1 min at 30% B. The electrospray source capillary voltage was 3500 V, and the ion source parameters for positive ion mode were: gas flow rate 16 L/min at 190 °C, sheath gas pressure 20 PSI at 350 °C, and nozzle voltage 1300 V.

The mobile phase for the negative ion mode analysis consisted of buffer A (0.5 mM ammonium fluoride and 10 mM ammonium acetate in water) and B (acetonitrile: IPA (50:50 v/v)). The gradient elution (19.1 min) at a flow rate of 0.3 mL/min was 10% B for 4 min, 85% B from 4 to 6.2 min, 95% B maintained from 6.2 till 10.2 min, and changed to 10% B at 10.4–14.4 min, 95% B from 14.4 to 16.2 min, maintained till 16.4 min with re-equilibration from 16.4 to 19.1 min at 10% B. The ESI source capillary voltage was 3000 V, and the ion source parameters for negative ion mode were: gas flow rate 14 L/min at 190 °C, sheath gas pressure 25 PSI at 400 °C, and nozzle voltage 1500 V.

Commercial ^13^C isotopically labeled standards for gangliosides are not commercially available. Protocols for ^13^C_18_ labeled GM1 and GM3 gangliosides in-house synthesis are in the supporting information and respective mass spectra in Figure S3. We used the labeled GM3 internal standard to determine the concentration of all gangliosides, except for GM1, determined using the corresponding labeled GM1 internal standard. Respective response factors (RF) to the labeled GM3 were calculated for all gangliosides. We processed raw data in Skyline (Version 20.1.0.76, MacCoss Lab., UW). All concentrations are the average of technical duplicates relative to the ACTB level. The SRM library is shown in Table S2. Chromatograms for all ganglioside species and internal standards are shown in Figure S4a.

### Protein Extraction and Enzymatic Proteolysis

After lipid extraction, the dried protein pellet with a glass bead was powdered (4 m/s, 10 s, two cycles with 10 s inter-time, BeadBlasterTM 24, Benchmark), solubilized in the ammonium bicarbonate (AmBic) buffer (50 mM) with sodium deoxycholate (5 mg/mL),^23^ vortexed (10 s, 2000 rpm, VELP Scientifica), mixed (10 min, 2035 rpm, HeidolphTM MultiReax), and sonicated (1 min, 80 kHz, Elmasonic P, Elma Schmidbauer GmbH). The total protein concentration was adjusted to 0.5 μg/μL by adding the AmBic buffer. Samples were centrifuged (1 min, 12300 RCF, Micro-Star 12, VWR, Radnor, Pennsylvania), and the volume of 60 μL (equivalent to 30 μg of total protein) was used to reduce (20 mM DTT in 2.5 mM AmBic; 10 min; 95 °C) and alkylate (40 mM IAA in 2.5 mM AmBic; 30 min; ambient, in the dark) proteins. The remaining volume of individual CO homogenates was pooled into a quality control (QC) sample. Identical to the analysis of individual COs, we used 60 μL aliquots of the QC sample (30 μg of protein). Trypsin was added in the ratio of 1:60 (enzyme: total protein content, w/w), and the Parafilm sealed samples were incubated (37 °C; 16 h; gentle shaking). The trypsin digestion efficacy was tested in QC samples after 2, 4, and 16 h (Figure S5).

The isotopically labeled (SIL) synthetic peptides were added (sample conc. ≈ 260 nmol/L) before quenching the digestion with 200 μL of 2% formic acid (FA). Samples were centrifuged (5 min, 12300 RCF), and the supernatant was loaded on the mixed-mode cartridge (Oasis PRiME HLB - 30 mg, Waters Corp. Milford, Massachusetts) for solid-phase extraction (SPE). Peptides were washed with 2% FA and eluted with 500 μL of 50% acetonitrile (ACN) with 2% FA, and the samples were dried in SpeedVac. SIL standard peptides (ST) response in the QC sample before and after the SPE was compared to determine the SPE recovery for tryptic peptides: peak area of ST(before SPE)/peak area of ST(after SPE) × 100. The average SPE recovery was 87% for all 14 quantifier proteotypic peptides (Figure S6a and Table S3).

### Mass Spectrometry Protein Assays and Data Processing

Dried SPE-purified peptides were reconstituted in 15 μL of 5% ACN with 0.1% FA. The QC sample homogenates with 40 μg total protein were reconstituted in 60, 40, and 20 μL to load 2, 3, and 6 μg total protein equivalent to UHPLC-SRM, respectively (Figure S6c,d). Peptides were analyzed in positive ion detection mode using the same UHPLC-MS system as for ganglioside assays. A sample volume (3 μL, equivalent to 6 μg of total protein) was injected into the C18 analytical column (Peptide CSH 1.7 μm, 2.1 × 100 mm^2^, Waters Corp., Milford, Massachusetts). The mobile phase flow rate was 0.3 mL/min; buffer A (0.1% FA) and buffer B (0.1% FA in 95% ACN). Linear gradient elution: initial 5% B; 25 min 30% B; 25.5 min 95% B; 30 min 95% B; and from 31 to 35 min with 5% B. The ESI source temperature was 200 °C, and the capillary voltage was 3500 V.

SRM protein assays were designed utilizing the neXtProt database (online, www.nextprot.org) to select proteotypic peptides (2–4 per protein), preferably with experimental evidence in the PeptideAtlas. SRM library (3–4 transitions per proteotypic peptide) was selected in the SRMAtlas (www.srmatlas.org), (Figure S2). The dwell time (10 ms) and a cycle time (<1 s) allowed for up to 100 transitions in every acquisition method. We tentatively identified peptides in QC samples using a retention time prediction model and verified the identifications using isotopically labeled synthetic analogues. We used a dynamic SRM (dSRM) mode with a 2 min-wide window centered at a peptide experimental retention time in the QC sample. We relatively quantified target proteins preferably using >5 *y*-ions with peak area >10 000 and reproducible response across technical duplicates (% coefficient of variation (CV) < 15), as shown in Figure S2. The dSRM assay included 251 transitions to monitor 41 unique peptides of 18 proteins (Table S4). The lowest total protein content in analyzed COs (*n* = 24) was 30 μg.

Data were processed in Skyline and manually inspected, Figure S4b. A single quantifier transition (Table S4) was used to determine relative concentrations (light peptide peak area/ST peptide peak area × ST peptide concentration). The protein levels in individual samples are reported as an average of technical duplicates normalized to ACTB levels.

### Ganglioside and Protein Assays Validation

Detailed information on assay validation is described in the Supporting Information: ganglioside assay validation and protein assay validation. For gangliosides, 10-point matrix-matched calibration curves were prepared and analyzed (Figure S7 and Table S5). Precision was <12.1% of %CV (Table S5b), ganglioside recovery was high (>82.3%), and matrix effects were negligible (Table S6). For proteins, 10-point calibration curves were prepared and analyzed (Figure S8 and Table S7) with *R*^2^ = >0.99 linear response range 1.02–81.25 nM for SOX2, 1.02–1300 for ACTB, GAPDH, and 1.02 or 5.08–325 nM for other proteins. The matrix effects were moderate, on average 32% (Figure S6b and Table S3), and signal reproducibility in the sample matrix was <11 %CV (Table S3).

### Data Analysis and Visualization

Cluster analysis for protein and lipid markers was prepared in MataboAnalyst 5.0 (online, https://www.metaboanalyst.ca/ (2021)). The graphs were prepared using GraphPad Prism version 8.0.2 for Windows, GraphPad Software, California (www.graphpad.com). Figures 1–4a, S1, S2, S4, S6, S10, and S11 were created with BioRender.com.

**Figure 2:**
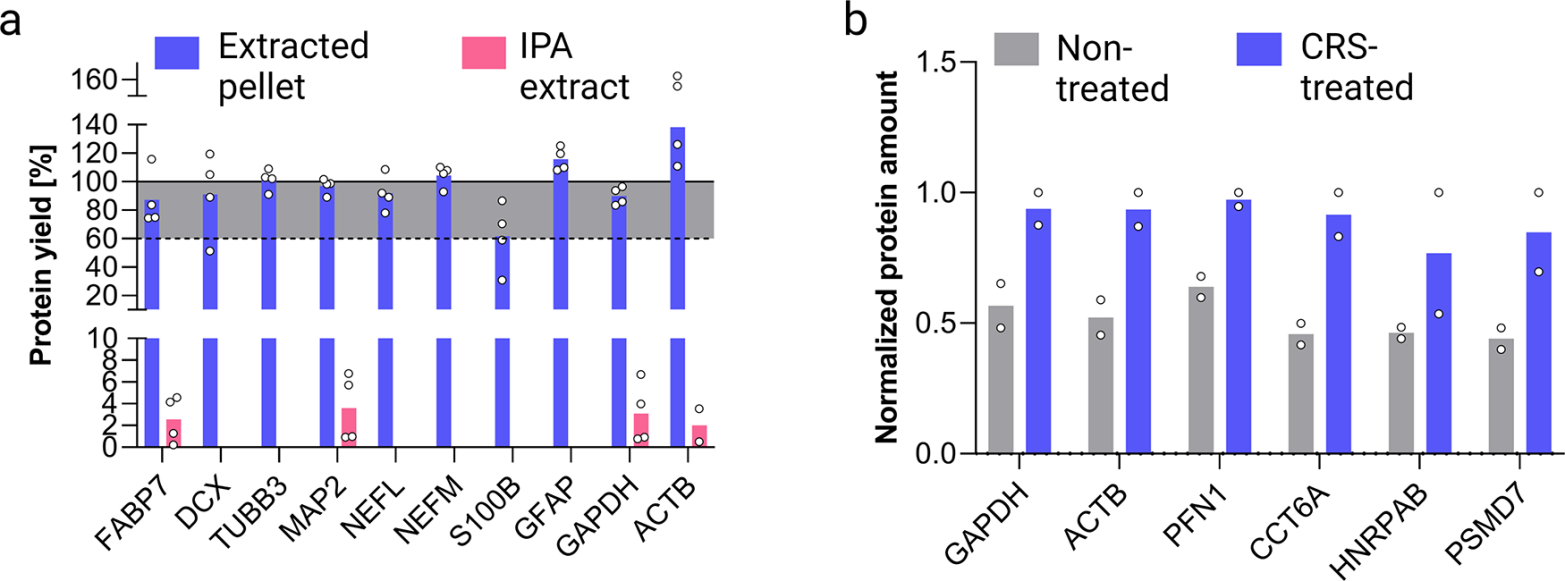
Analytical figures of merit of the mass spectrometry-based workflow. (a) Loss of targeted proteins to isopropanol (IPA) after lipid extraction. The protein content in the residual pellet and the IPA extract was compared to the total protein amount in the homogenate not subjected to IPA extraction. The result is expressed as protein yield in %. Four target proteins were detected in the IPA extract, and the protein loss was <5%. (b) Geltrex removal using cell recovery solution (CRS). Housekeeping protein levels were analyzed in 30 μg of processed cerebral organoid total protein (*n* = 2). On average, 2-fold higher levels were found in CRS-treated organoids relative to untreated.

**Figure 3:**
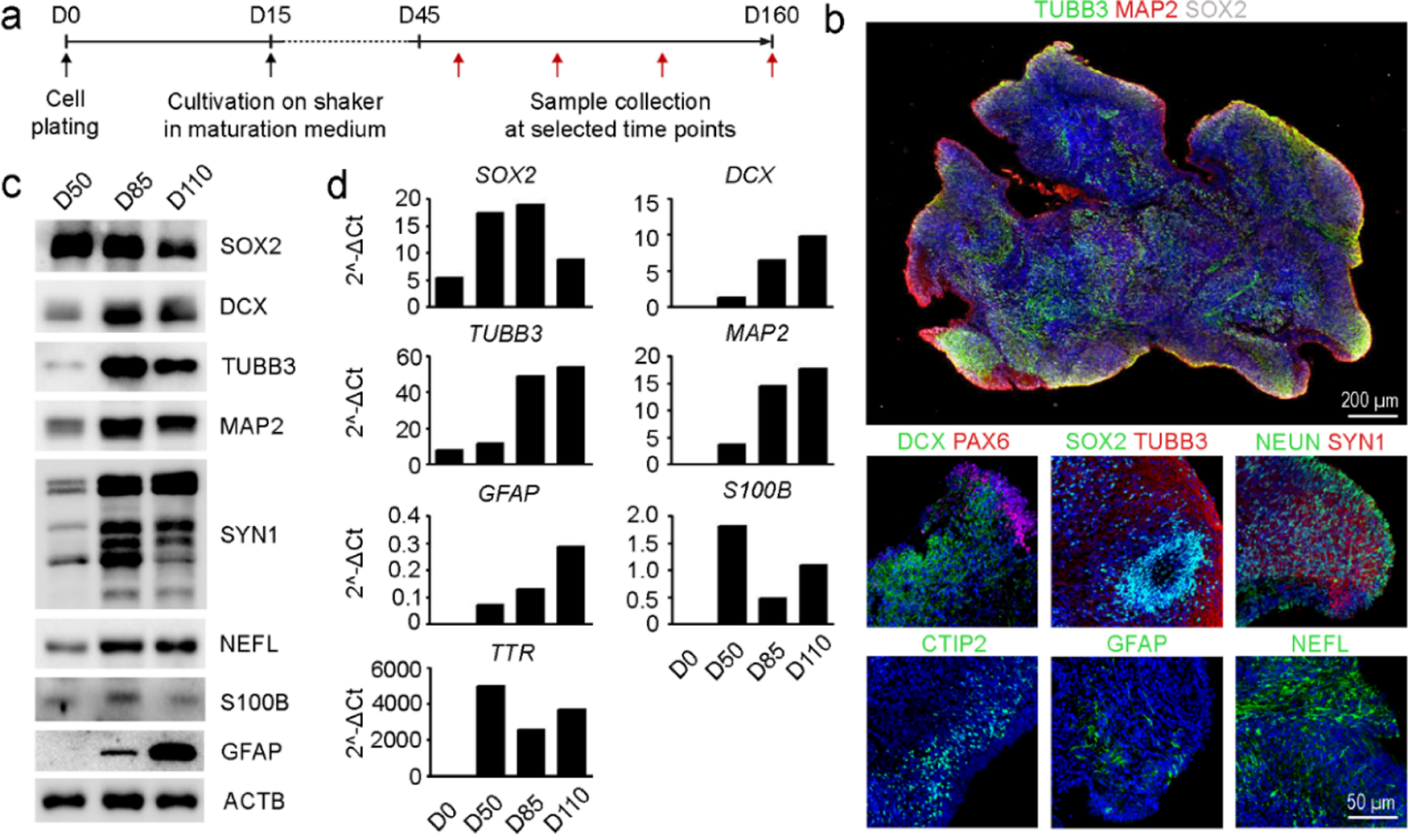
Cerebral organoid differentiation and development characterized by immunoaffinity and qPCR assays. (a) Timeline of cerebral organoid differentiation. (b) Morphology of the cross section of mature organoids cultivated for 85 days visualized by indirect immunofluorescent staining—scale bars: top 200 μm, bottom 50 μm. (c) Immunoblotting and (d) qPCR assays show cell type-specific markers in organoids collected on days 50, 85, and 110 of differentiation, *n* = 5–7 (pooled) per time point. Cell-specific markers for neural stem cells (SOX2, PAX6), mature and immature neurons (NEUN, MAP2, and TUBB3, DCX, respectively), synaptic junctions (SYN1), neurofilaments light (NEFL), first cortical layer neurons (CTIP2), and astrocytes (S100B, GFAP) were detected. ACTB served as the loading control for the immunoblotting assay, and qPCR data were normalized to GAPDH levels.

**Figure 4:**
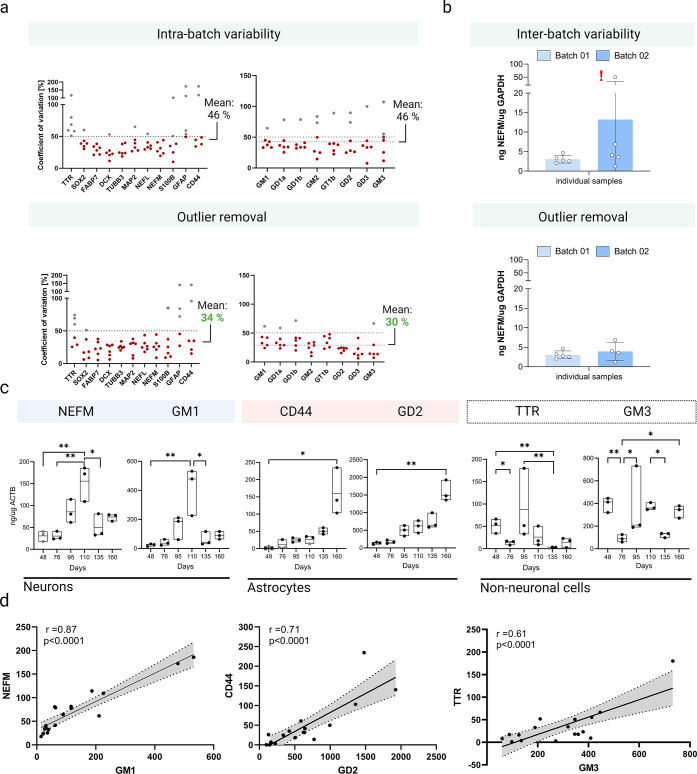
Single cerebral organoid characterization by mass spectrometry assays for cell-specific protein and lipid markers. (a) Intra-batch variability of target proteins and lipids in organoids from timeline experiment before and after the application of outlier removal. (b) Interbatch variability of neuronal population in organoids from two cultivation batches. (c) Single-organoid time trends in levels of specific traits (after outlier removal) for neurons (NEFM, GM1), astrocytes (CD44, GD2), and non-neuronal cells (TTR, GM3), *n* = 3 per time point. A significant increase in neuronal and astrocyte populations was visible (*p-value < 0.05, **p-value < 0.01). (d) Correlation plots for protein and lipid markers for neurons (NEFM, GM1), astrocytes (CD44, GD2), and non-neuronal cells (TTR, GM3).

## Results and Discussion

### Characterization of Cell-Specific Markers via qPCR, Immunoblotting Assay, and Indirect Immunofluorescence

We used a protocol modified by Lancaster et al.^2^ to differentiate COs^2^ (Figure 3a). An average D85 CO can range from 3 to 5 mm in diameter and consists of 2.5 million cells (Figure 3b). The COs’ morphology on D85 was characterized by indirect immunofluorescence. The cell-specific marker expression was assessed via WB (Figure 3c) and qPCR (Figure 3d), pooling 5–7 COs per assay. Consistently with the previous reports,^2,24^ we demonstrate the expression of markers for neuroectodermal cells (SOX2, PAX6), neurons (MAP2, TUBB3, DCX, and NEUN), deep-layer neurons (CTIP2), synaptic junctions (SYN1), neurofilaments (light chain, NEFL), and astrocytes (S100B, GFAP). The protein expression of SOX2 reached a maximum on D50 and later declined. Neuronal (i.e., MAP2, DCX, and TUBB3) markers and astrocytic GFAP were at the maximum level on D110. Detailed information on cultivation and analysis is described in the Supporting Information.

### Extraction of Gangliosides and Proteins from Cerebral Organoids for Mass Spectrometry Assays

COs were CRS-treated to remove the Geltrex matrix before MS analysis (Figure 1). Isopropanol (IPA) was added to homogenized COs to extract gangliosides and precipitate proteins. The protein loss due to IPA extraction was <5% (Figure 2a). We compared housekeeping protein (HKP) levels in CRS-treated and nontreated COs to assess the efficiency of matrix removal. HKP levels (Figure 2b) and gangliosides’ internal standards’ signals (Figure S9) in CRS-treated samples were up to 2-fold higher than in nontreated samples. The enriched cellular proteins and gangliosides in CRS-treated COs improved assay sensitivity.

### Heterogeneity in Cerebral Organoids

We profiled cell populations using protein and lipid markers in individual COs after 48, 76, 95, 110, 135, and 160 days of differentiation (Figures 4, S10, and S11). COs were analyzed individually at each time point (*n* = 4) to remove one outlier per time point (*n* = 3). Detailed information on heterogeneity is described in the Supporting Information: Heterogeneity in individual cerebral organoids. The outlier removal reduced CV within the batch from 46 to 34 and 46 to 30% for protein and lipid markers, respectively (Figure 4a). After outlier removal, we observed stronger correlations between markers, such as GD2 vs CD44 (before *r* = 0.45, *p* = 0.0003 and after outlier removal *r* = 0.71, *p* < 0.0001) and GM3 vs TTR (before *r* = 0.32, *p* = 0.0042 and after outlier removal *r* = 0.61, *p* < 0.0001); data not shown.

In addition, we analyzed two batches of COs (*n* = 5) derived from the same cell line and harvested at identical time points to perform an interbatch variability analysis. The interbatch variability was reduced substantially after outlier removal (Figure 4b), which is not feasible in pooled samples.

The protein and lipid markers panel were characterized in individual COs, and results are shown after outlier removal (*n* = 3 per time point) (Figures 4c and S11).

### Cell-Specific Protein Expression in Cerebral Organoids

Cell markers for NSCs, radial glial cells, neurons, astrocytes, and the ubiquitously present housekeeping proteins were relatively quantified in COs. The total cell mass estimated using HKP (i.e., GAPDH and ACTB) levels reached a maximum between D76 and D95 (Figure S12). On D48, SOX2 was the most abundant marker in COs and later downregulated (Figure S11). In parallel with the SOX2 decline, the expression of RGCs marker FABP7 increased until D76 and later remained steady (Figure S11). TTR expression attributed to the choroid plexus epithelial cells reached a maximum in D95 (Figure 4c). Neuronal markers’ expression increased from D76 until D110, followed by a steady state or decline, while astrocyte markers’ expression increased until D160 (Figures 4c and S11). Neuron-specific proteins DCX, TUBB3, MAP2, NEFL, and NEFM, emerged early (D48), culminated on D110, and later declined, except for MAP2 (Figure S11). The mature neurons’ marker SYN1 emerged from D95 (Figure S11). The astrocytic markers (S100B, GFAP, and CD44), negligibly expressed on D48, gradually increased until D160 (Figures 4c and S11).

We characterized some protein markers using WB and qPCR assays to align with the reported LC-MS-based workflow (Figure 3c,d) and previous studies demonstrating the development of neuron and astrocyte populations to mimic the neurogenesis in vivo.^10,24^ WB and MS assays identically show the highest NSCs’ population (SOX2) at an early stage (48D) of COs’ proliferation (Figures 3c and S11). Temporal trends of neuronal markers (i.e., DCX, TUBB3, MAP2) and astrocytic markers (i.e., S100B, GFAP) determined by WB and qPCR mainly agreed with MS-based assays, except for qPCR assessed S100B and GFAP showing an earlier onset (Figure S13).

However, only a limited number of cell-specific markers can be determined in pooled COs by immune-based assays^25−27^ without assessing the variability in individual COs. On the other hand, the SRM assay allows the characterization of multiple analytes in a single organoid with high specificity and multiplexing capability for protein quantification.

### Membrane Glycosphingolipids in Cerebral Organoids

Apart from gangliosides, the lipid extract was utilized to monitor other major lipid species. We characterized 351 lipid species from over 24 lipid classes composed of cholesterol, phospholipids, lysophospholipids, ceramides, sphingolipids, triacylglycerols, and carnitines (Figure S14). Gangliosides GM1, GM2, GM3, GD1a, GD1b, GD2, GD3, and GT1b are abundant in the nervous tissue.^28^ The monosialo GM3 and disialo GD3 represent NSCs markers.^29^ GD3 was the most abundant ganglioside in the COs (Figure S11), and the levels of GD3 and GM3 remained steady at all time points, indicating high NSC reserve even at a late stage of CO proliferation, an analogy with mature brain tissue.^30^ GD3 interacts with the epidermal growth factor receptor (EGFR) and induces neural precursor cell differentiation and neurite formation.^31^ We observed a progressive increase in complex neuronal gangliosides from D48 until D110, followed by a decline in D135 and D160 (Figures 4c and S11). The biosynthesis switch possibly indicates the neuronal differentiation stage from GD3 and GM3 to complex neuronal gangliosides (i.e., GD1a, GD1b, GT1b, and GM1), involved in signaling neurogenesis and astrocytogenesis.^32^ GM2 and GD2 have been associated with astrocytes.^33,34^ GD2 and GM2 levels increased gradually in COs, with a maximum at D160, paralleled by astrocyte protein markers, alluding to their possible colocalization in astrocytes (Figures 4c,d, S11, and S15 and Table S11).

## Conclusions

COs have been increasingly used as a brain model.^2^ However, the 3D cell cultures suffer from the “batch effect” caused by variations in the differentiation, morphology, and cell composition.^5^ High intra- and interbatch differences limit the reproducibility of experiments and may induce false discoveries.

We developed a mass spectrometry-based profiling of cell-specific proteins (Table S1) and lipid traits with high selectivity, sensitivity, and reproducibility in a single CO (Figures 4c and S11). LC-MS can characterize a single cerebral organoid and may be applied repeatedly using different LC separation conditions and SRM assays to profile hundreds of analytes quantitatively. Pre-analytically, we removed the organoid matrix to mitigate a nonspecific binding of small molecules and peptides to cell culture media,^35^ reducing interferences with LC-MS analysis^36^ (Figure 1). We presented a systematic workflow for relative protein quantification (Figure S2). We demonstrated that the characterization of individual COs using a panel of cell-specific protein markers and lipid traits could be used to reduce intra-batch and interbatch variability post-analytically by discarding results from abnormally differentiated cerebral organoids. However, our method requires analyzing 3–5 COs per group/condition to identify outliers, which may lead to extensive cell culture.

Our study’s protein and lipid traits characterized for various cell populations demonstrate the requisite complexity of COs to mimic neurodevelopment and aging features. Despite the heterogeneity, our characterization protocol shows the potential of COs as a model for the neurobiology of human neurological disorders.

## Supporting Information Available

Additional experimental details on chemicals and reagents; cerebral organoid cell culture; RNA isolation, cDNA synthesis, and real-time quantitative PCR (qPCR) assay; immunoblotting assay for cell-specific markers; indirect immunofluorescent staining; ganglioside and protein assay validation; cluster analysis of cell-specific protein markers and glycosphingolipid traits; assessment of heterogeneity in individual cerebral organoids; major lipids characterization; localization of neuronal (DCX, TUBB3, MAP2, NEFL, NEFM, SYN1) and astrocytic (S100B, CD44, GFAP) markers and housekeeping proteins (ACTB, GAPDH) (Figure S1); protein identification and relative quantification workflow (Figure S2); mass spectra for the 13C labeled GM3 and GM1 synthesized in-house (Figure S3); representative chromatograms for lipids and proteins (Figure S4); trypsin digestion efficiency after 2, 4, and 16 h of incubation (Figure S5); analytical parameters of proteomic protocol (Figure S6); calibration curves for ganglioside isotopically labeled standards (Figure S7); calibration curves of synthetic isotopically labeled peptide internal standards used for protein assays (Figure S8); cell recovery solution (CRS) treatment to remove Geltrex sample matrix (Figure S9); representative chromatograms of a peptide and a ganglioside in blank, QC, CO D48, and CO D160 samples (Figure S10); the time trends of neuron-specific markers (DCX, NEFL, TUBB3, MAP2, SYN1, GD1a, GD1b, and GT1b); a marker of choroid plexus epithelial cells (TTR); NSCs marker (GD3); RGCs marker (FABP7); and astrocyte-specific markers (S100B, GFAP, and GM2) (Figure S11); housekeeping proteins ACTB and GAPDH represent the total cell mass during cerebral organoid proliferation (Figure S12); temporal trends of selected protein markers comparing reference methods (i.e., WB, qPCR) with MS assays (Figure S13); the time trends of major lipids in cerebral organoids (Figure S14); the cluster analysis of proteins and gangliosides at different stage of neurogenesis (Figure S15); variability scores the sum of lipid markers levels, the sum of protein markers levels, and neurons to glia ratio of individually profiled cerebral organoids (Figure S16); protein markers profiled in cerebral organoids (Table S1); selected reaction monitoring (SRM) library of precursor/product ion transitions for analyzing gangliosides in positive and negative ion detection modes (Table S2); synthetic isotopically labeled peptides for protein assays: signal reproducibility, recovery, and matrix effects (Table S3); SRM library for synthetic isotopically labeled peptides for protein assays (Table S4); calibration curve for ganglioside assays (Table S5); ganglioside internal standards: signal reproducibility, recovery, and matrix effects (Table S6); calibration curve parameters for protein assays (Table S7); the variability in quantitation of protein and lipid markers before (a) and after (b) removing outlier cerebral organoids (Table S8); the algorithm to assess heterogeneity in cerebral organoids (Table S9); a score to assess potential sources of heterogeneity in cerebral organoids (Table S10); correlation coefficient for the protein and lipid markers (Table S11); list of qPCR primers (Table S12); and proteomics protocol reproducibility (Table S13) (PDF)
