# Supplementary material for: Single Cerebral Organoid Mass Spectrometry of Cell-Specific Protein and Glycosphingolipid Traits
Source: Anal Chem. 2023 Feb 1;95(6):3160–7. doi: 10.1021/acs.analchem.2c00981 (PMC10016744; doi:10.1021/acs.analchem.2c00981)
Supplement: Supplementary file 1 — ac2c00981_si_001.pdf [file ac2c00981_si_001.pdf]

Supporting Information

Single cerebral organoid mass spectrometry of cell-specific protein and glycosphingolipid traits

Markéta Nezvedová[1‡], Durga Jha[1‡], Tereza Váňová[2,3], Darshak Gadara[1], Hana Klímová[2], Jan Raška[2], Lukáš Opálka[4], Dáša Bohačiaková[2,3], Zdeněk Spáčil[1*]

[1]RECETOX, Faculty of Science, Masaryk University, Brno, 625 00 Czech Republic

[2]Dept. of Histology and Embryology, Faculty of Medicine, Masaryk University, Brno, 625 00 Czech Republic

[3]International Clinical Research Center (ICRC), St. Anne's University Hospital, Brno, 656 91 Czech Republic

[4]Dept. of Chemistry, Faculty of Pharmacy, Charles University, Hradec Kralove, 500 05 Czech Republic

‡ Equal contribution

* Corresponding Author:

Dr. Zdenek Spacil

Masaryk University, Faculty of Science, RECETOX

Kamenice 753, 625 00 Brno, Czechia

Phone: +420 549 49 7989

E-mail: spacil@u.washington.edu or spacil@recetox.muni.cz

Table of Contents

Experimental methods: 3

Chemicals and Reagents 3

Cerebral organoids cell culture 3

RNA isolation, cDNA synthesis, and Real-Time quantitative PCR (qPCR) assay 4

Immunoblotting assay for cell-specific markers 4

Indirect immunofluorescent staining 4

Synthesis of isotopically labeled internal standards 5

Ganglioside assay validation 6

Protein assay validation 6

Cluster analysis of cell-specific protein markers and glycosphingolipid traits 7

Heterogeneity in individual cerebral organoids 7

Major lipid characterization 7

Supplementary figures: 9

Figure S1. 9

Figure S2. 10

Figure S3. 11

Figure S4. 12

Figure S5. 14

Figure S6. 15

Figure S7. 16

Figure S8. 17

Figure S9. 19

Figure S10. 20

Figure S11. 21

Figure S12. 22

Figure S13. 23

Figure S14. 24

Figure S15. 25

Figure S16. 26

Supplementary tables 27

Table S1. 27

Table S2. 28

Table S3. 29

Table S4. 30

Table S5. 39
Table S6. 40
Table S7. 41
Table S8. 42
Table S9. 43
Table S10. 44
Table S11. 45
Table S12. 47
Table S13. 48

Experimental methods:

Chemicals and Reagents

Synthetic isotopically labeled (SIL) peptide standards (SpikeTides_L crude) were from JPT Peptide Technologies Inc. (Acton, MA, USA). Isotopically-labeled ganglioside (GS) internal standards (GM1 and GM3) were synthesized by Lukas Opalka (Faculty of Pharmacy, Charles University). Pierce BCA Protein Assay Kit reagents were from Ther-moFisher Scientific, Waltham, MA, USA. The ultrapure water was prepared in the purification system (arium® Comfort System, Sartorius).

Cerebral organoids cell culture

Undifferentiated human iPSCs (line M8) were propagated as a feeder-free culture on Vitronectin (ThermoFisher Scientific) coating in Essential 8™ Medium (ThermoFisher Scientific) as previously described[23]. Cells were passaged every 4-5th day using 0.5mM EDTA solution. Differentiation to cerebral organoids was performed according to Lancaster and Knoblich 2014[24]. Undifferentiated iPSCs were harvested using Accutase (ThermoFisher Scientific), and 9000 cells per well were plated into a non-adhesive V-shaped 96-well plate (coated with poly-2-hydroxyethyl methacrylate, Sigma-Aldrich). Cells in 96-well plates were spun down at 200 rpm for 6 min. The first differentiation medium (I) was used until day six of cultivation and consists of DMEM/F12 supplemented by 20% KnockOutTM Serum Replacement, 3% Embryonic Stem Cell Fetal Bovine Serum Qualified, 1% GlutaMAX, 1% non-essential amino acids (all from ThermoFisher Scientific), 1% ZellShield (Minerva Biolabs, Berlin, Germany), β-mercaptoethanol (100µM; Sigma-Aldrich) and human recombinant FGF2 (4 ng/ml; ThermoFisher Scientific). Cells were cultivated for four initial days with Rock inhibitor (20µM; Selleckchem, Houston, TX, USA). On day six, cells were transferred into non-adhesive 48-well plates and grown in the second differentiation medium (II) consisting of DMEM/F12 with 1% GlutaMAX, 1% non-essential amino acids, 1% Zell-Shield, heparin (1 µg/ml; Sigma-Aldrich) and 1% N2 (ThermoFisher Scientific). On day 11, organoids were embedded into 17 µl of GeltrexTM containing extracellular proteins purified from murine Engelbreth-Holm-Swarm tumor (ThermoFisher Scientific) and cultivated in a 6 cm petri dish in the third differentiation medium (III) consisting of DMEM/F12 and Neurobasal 1:1 with 1% GlutaMAX, 0.5% non-essential amino acids, 1% ZellShield, 100 µM β-mercaptoethanol, 2.5 µg/ml Insulin (Sigma-Aldrich), 1% B27 without vitamin A (ThermoFisher Scientific) and 0.5% N2. Since day 15, organoids were cultivated on an orbital shaker in the fourth differentiation medium (IV) consisting of DMEM/F12 and Neu-robasal 1:1 with 1% GlutaMAX, 0.5% non-essential amino acids, 1% ZellShield, 100µM β-mercaptoethanol, 2.5 µg/ml Insulin, 1% B27 (ThermoFisher Scientific) and 0.5% N2.

For SRM-MS assays, COs were harvested after 48, 76, 95, 110, 135, and 160 days (+/- 2 days) of proliferation (D48, D76, D95, D110, D135, and D160), four biological replicates per time point and analyzed individually.

For qPCR assays and Western blotting (WB), COs were harvested at representative time points (D50, D85, and D110), and 5-7 pooled COs were analyzed per each time point.

RNA isolation, cDNA synthesis, and Real-Time quantitative PCR (qPCR) assay

Total RNA was isolated from undifferentiated iPSCs on day 0 (D0) and differentiating COs using the RNA Blue (Top-Bio, Prague, Czechia). The concentration and purity of isolated RNA were determined using NanoDrop 1000 (ThermoFisher Scientific). According to the manufacturer's instructions, the RNA was transcribed to cDNA using Transcriptor First Strand cDNA Synthesis Kit (Roche, Basel, Switzerland). qPCR was performed from the cDNA samples using LightCycler® 480 SYBR Green I Master kit (Roche) on LightCycler 480 II (Roche). Samples were analyzed in technical triplicates, and results were normalized to respective glyceraldehyde 3-phosphate dehydrogenase (GAPDH) gene values. Primers are listed in Supplementary **Tab. S12**.

Immunoblotting assay for cell-specific markers

WB was performed as described previously in Fedorova et al., 2019[25]. Briefly, harvested organoids were lysed in 50mM Tris-HCL with 1% SDS and 10% glycerol. Protein concentrations were measured using DC Protein Assay (Bio-Rad, Hercules, CA, USA) and adjusted to the same level. Proteins were separated using a 10% SDS-PAGE and transferred onto the PVDF membrane (Merck Millipore, Darmstadt, Germany). Membranes were blocked in 5% skimmed milk in 4mM Tris-buffered saline with 0.05% Tween 20 (TBS-T) and incubated with primary antibodies (4 °C; overnight), followed by incubation with horseradish conjugated secondary antibodies (ambient temperature; 1 h). Proteins were visualized by ChemiDoc (Bio-Rad) using Amersham ECL Prime western blotting Detection Reagent (GE Healthcare Life Sciences, Chicago, IL, USA). Following primary antibodies were used in the immunoblotting analysis: DCX (sc-271390, Santa Cruz Biotechnology, Dallas, TX, USA); S100B (ab11178, Abcam, Cambridge, GB); SOX2 (4900S, CST, Danvers, MA, USA); TUBB3 (5568S, CST); MAP2 (8707T, CST); NEFL (2837S, CST); SYN1 (5297S, CST); GFAP (12389P, CST) and ACTB (3700S, CST).

The performance highly depends on the antibodies' quality. The whole membrane should be incubated for accurate results with the primary antibody to reveal protein isoforms, post-translational modifications, or antibody non-specificity. In our laboratory, a horseradish peroxidase-conjugated secondary antibody was used for detection, so only three specific proteins can be analyzed in a 30 µg of organoid protein. This method can be modified by using fluorescent secondary antibodies, whereby two (or more) primary antibodies derived from different animals can be applied to the membrane; in this case, up to six proteins can be detected from the 30 µg of organoid protein. Another commonly used approach to spare the protein sample is to cut the membrane according to the size of the protein and incubate the different parts of the membrane in antibodies individually. It is necessary to examine proteins with different molecular weights and have well-validated primary antibodies, and then 6-9 different proteins can be detected.

Indirect immunofluorescent staining

COs were fixed in 3.7% paraformaldehyde (P-LAB, Prague, Czechia) and embedded into 3% agarose (Sigma-Aldrich) before paraffin embedding. Thin sections (2 µm) were deparaffinized in xylene and rehydrated through a descending series of ethanol. Slices were treated with antigen retrieval (pH6, DAKO, Carpinteria, CA, USA) for 20 min at 98 °C, permeabilized in 0.2% Triton-X (Sigma-Aldrich) in PBS, and blocked in 2% normal goat serum (Sigma-Aldrich) in permeabilization solution. Sections were incubated with primary antibodies diluted in blocking solution (4 °C; overnight) and with fluorophore-conjugated secondary antibodies (ambient temperature; 1h) Alexa Fluor A21202 and A10042, Thermo Fisher). Nuclei were visualized by 4',6-diamidino-2-phenylindole (DAPI) using a fluorescent microscope Tissue FAXS (Tissue Gnostics GmbH Vienna, Austria). In addition to the above listed primary antibodies PAX6 (60433S, CST); NEUN (24307S, CST); and CTIP2 (12120S, CST) antibodies were used for immunofluorescent staining.

Synthesis of isotopically labeled internal standards

All chemicals for the synthesis (including lactosyl sphingosine, $^{13}C_{18}$ stearic acid, N-(3-dimethylaminopropyl)-N′-ethylcarbodiimide, cytidine-5′-monophospho-N-acetylneuraminic acid sodium salt, sodium taurodeoxycholate hydrate, α-2,3-sialyltransferase from Pasteurella multocida, alkaline phosphatase from bovine intestinal mucosa, sphingolipid ceramide N-deacylase from Pseudomonas sp., N-hydroxysuccinimide) were obtained from established suppliers (Sigma-Aldrich, Schnelldorf, Germany; Merck, Darmstadt, Germany) and were used as received. MonosialoGSs GM1 was obtained from Matreya, State College, PA, USA. TLC was performed on Merck aluminum sheets with silica gel 60 F254. Merck Kieselgel 60 (0.040–0.063 mm) was used for column chromatography. Prefilled Supelclean ENVI – 18 SPE Tubes were received from Supelco, Bellefonte, PA, USA. For TLC visualization was used ammonium molybdate with ceric sulfate in sulfuric acid.

Chemical synthesis of $^{13}C_{18}$ labeled ganglioside GM3

GM3 The amount of 5.3 mg (0.0085 mmol) of lactosyl sphingosine was mixed with 3.3 mg (0.0109 mmol) of $^{13}C_{18}$ stearic acid and 3.5 mg (0.0259 mmol) of 1-hydroxybenzotriazole, dried on the vacuum, dissolved in 0.5 mL of dry DCM and 0.75 mL of dry MeOH and cooled with ice to 0 °C. 3 uL (0.0170 mmol) of N-(3-dimethylaminopropyl)-N′-ethylcarbodiimide were added, and the temperature was allowed to increase to RT. The reaction mixture was stirred at RT under argon atmosphere for 24 hours. After 24 hours, the resulting suspension was dissolved in excess of MeOH and the solution was evaporated with silica. The column chromatography CHCl3/MeOH/2.5M NH4OH 60:40:10 provided 7.4 mg (96%) of $^{13}C_{18}$ labeled lactosylceramide as a white solid. The product identity was confirmed using MS: C3013C18H91NO13, [M+Na]+ calculated 930.70, measured 930.72; [2M+Na]+ calculated 1838.41, measured 1838.84. In total, 7.4 mg (0.0081 mmol) of lactosylceramide from the previous reaction was mixed with 5.2 mg (0.0082 mmol) of cytidine-5′-monophospho-N-acetylneuraminic acid sodium salt and dissolved in 2 mL of TRIS buffer (pH 8, 100 mM). 4.3 mg (0.0082 mmol) of sodium taurodeoxycholate was added into the resulting suspension, and the suspension was shortly sonicated to yield a cloudy solution. Using TRIS buffer, the enzyme α-2,3-sialyltransferase from Pasteurella multocida (1 UN) was added into the solution, followed by the addition of 1 mg (10.5 µmol) of MgCl2 and 100 µL of the alkaline phosphatase from bovine intestinal mucosa in TRIS buffer (the equivalent of 100 DEA units). The reaction mixture was stirred in the incubator at 37 °C and monitored using MS. After 6 hours, the MS showed complete consumption of lactosylceramide, and the reaction was quenched. The solvent was carefully evaporated, the residue was dissolved in MeOH and evaporated with silica. The product was purified using column chromatography CHCl3/MeOH/2.5M NH3 100:45:10 to yield 2.9 mg (30 %) of the $^{13}C_{18}$ labeled GSs GM3 as a white solid. The product identity was confirmed using MS: C4113C18H108N2O21, [M+Na]+ calculated 1221.79, measured 1221.90; [M-H]- calculated 1197.80, measured 1198.01.

Chemical synthesis of $^{13}C_{18}$ labeled ganglioside GM1

GM1 The amount of 5 mg (0.0032 mmol) of monosialoGSs GM1 (NH4+ salt) was dissolved in 1 mL of acetate buffer (pH 5.8, 100 mM), 11 mg (0.099 mmol) of CaCl2 and 4 mg (0.0077 mmol) of sodium taurodeoxycholate were added followed by the addition of sphingolipid ceramide N-deacylase from Pseudomonas sp. (0.25 UN). The reaction mixture was stirred at 37 °C in the incubator for 48 hours. After 48 hours, the resulting suspension was centrifuged (6000 RPM, 4 min) and the supernatant was collected. The solid residue was washed with 300 uL of water and centrifuged again. The supernatant was collected and added to the previous one. The combined supernatants were applied on the prefilled RP18 column and washed first with water, then with 65% MeOH and finally with 85% MeOH. This purification provided 1.8 mg (44%) of the lysoGSs GM1 as a white solid. The product identity was confirmed using MS: C55H97N3O30, [M-H]- calculated 1278.61, measured 1278.70. In total, 3 mg (0.0099 mmol) of $^{13}C_{18}$ stearic acid were mixed with 1.7 mg (0.0149 mmol) of N-hydroxysuccinimide and dried on the vacuum. 1 mL of dry DCM was added, and the reaction mixture was cooled with ice to 0 °C. 3.5 uL (0.0198 mmol) of N-(3-dimethyl aminopropyl)-N′-ethyl carbodiimide was added, and the temperature was allowed to increase to

RT. The reaction was stirred at RT under an argon atmosphere for 48 hours. After 48 hours, the reaction mixture was diluted with 2 mL of DCM, evaporated on silica, and purified by column chromatography Hex/EtOAc 3:1 with 0.5% AcOH to obtain 2.6 mg (66%) of succinimide-1-yl $^{13}C_{18}$ stearate. The product was used in the next reaction without further characterization. In total, 1.8 mg (0.0014 mmol) of lysoGSs GM1 was mixed with 1 mg (0.0025 mmol) of succinimide-1-yl $^{13}C_{18}$ stearate and dried on the vacuum. 250 uL of dry DMF and 1.2 uL (0.0070 mmol) of DIPEA were added, and the reaction mixture was stirred at RT under argon atmosphere for 48 hours. After 48 hours, DMF was evaporated and the residue was dissolved in MeOH and evaporated on silica. The crude product was purified using column chromatography, first CHCl3/MeOH 60:40, then CHCl3/MeOH/2.5M NH4OH 60:40:10 to yield 1.7 mg (77%) of $^{13}C_{18}$ labeled GSs GM1. The product identity was confirmed using MS: C5513C18H131N3O31, [M+H]+ calculated 1564.94, measured 1564.95.

Ganglioside assay validation

We prepared a ten-point matrix-matched calibration curve adding the isotope-labeled internal standards to the pooled lipid extract in concentrations from $3.e^{-3}$ to 50 µM for GM3 and from 3.e-5 to 0.6 µM for GM1. The four low-concentrated dilutions were analyzed in technical quadruplicates, and the six high-concentrated dilutions in triplicates, given biological material availability (**Tab. S5a**). The calibration curves for positive and negative ion detection modes are in **Fig. S7**. The linear regression correlation coefficients were >0.98. LOD was defined as S/N >3, and LOQ as the lowest concentration with CV <20 %. LOD and LOQ for individual GSs were corrected using respective response factors LOD_{GS} = (LOD_{GM3}*/RF_{GC}). The linear response range was 8.e-3 to 0.6 µM for GM1 and GM2 and from 8.e-3 to 50 µM for other GSs; Precision on QC samples (n=5) was between 1.8 to 12.1 % of the CV (**Tab. S5b**). Recoveries of GM3 and GM1 were 82.3 % and 105.1 %, respectively. Matrix effects determined at concentration 0.3 µM were negligible 108.9 % and 110.7 % for 13C_GM3 and 13C_GM1, respectively (**Tab. S6**).

Protein assay validation

We prepared a ten-point calibration in the range of expected sample concentrations determined in the QC sample (**Tab. S7a**). The calibration curves for quantifier peptides in positive ion mode are in **Fig. S8**. We calculated sample matrix LOD and LOQ for each protein using the standard deviation (STDEV) of first reproducibly (CV<20 %) detected quantifier transition of ST peptide (**Tab. S7b**):

LOD=3*STDEV/SLOPE(calibration curve)

LOQ=10*STDEV/SLOPE(calibration curve)

We assessed the QC samples' matrix effects, comparing ST peptide responses in the sample matrix after SPE with corresponding responses in the neat solution. The matrix effect was calculated: ST(matrix) peak area/ST(solution) peak area*100. The matrix effects were moderate on average 32 %, except for severe 83 % for S100B - peptide AMVALIDVFHQYSGR and 79 % for SOX2 – peptide LLSETEK (**Tab. S3**). However, quantifier peptides' responses were reproducible in neat solution (CV<18 %) and the sample matrix (CV<11 %) (**Tab. S3**).

We compared total protein concentrations in IPA extracted QC sample protein pellets with non-extracted QC sample protein pellets. In parallel, we analyzed the IPA extracts to detect potential protein losses. We tested the reproducibility of the protein pellet solubilization. To assess the protein extraction reproducibility, we dried equivalent aliquots of the QC sample (n=2) and performed the entire proteolytic protocol with an average CV<12 % for individual protein marker levels. The overall proteomics protocol reproducibility (CV, n=8), in QC samples without additional dry-down and reconstitution step, was on average, 11 %, from 3 % (GAPDH) to 21 % (NEFM) (**Tab. S13**).

Cluster analysis of cell-specific protein markers and glycosphingolipid traits

We grouped cell-specific proteins and gangliosides traits profiled in COs during early (D48, D76, D95; n=9) and late neurogenesis (D110, D135, D160; n=9). Similar to the previous reports[2,9,10,25], we observed the expected clustering pattern during the early cortical neurogenesis with main clusters: i) NSC, RGC, and peripheral tissue markers (SOX2, GM3, GD3, TTR)[11,29], ii) neuron traits (TUBB3, DCX, NEFL, NEFM, GM1, GD1a, GD1b, GT1b, GM2, GD2)[32], and iii) astrocyte traits (GFAP, S100B, CD44, MAP2)[25,33,34] (**Fig. S15a)**. The expression of cellular protein and ganglioside traits varied between early and late neurogenesis in COs. In the late neurogenesis, we report DCX and TUBB3 as markers of immature neurons, NEFL, NEFM, GD1b, GM1, GT1b, GD1a traits of mature neurons, GFAP and CD44 markers of mature astrocytes, and TTR with GM3 traits of peripheral tissue structures (**Fig. S15b)**. Major lipids in the organoid showed a trend like TTR, GM3, and GD3 (**Fig. S14**).

Our results showed parallel trends in neuronal protein markers (DCX, TUBB3, NEFL, NEFM) and gangliosides traits (GM1, GD1a, GD1b, and GT1b), and similarly for the astrocyte protein markers (S100B, GFAP, and CD44) and gangliosides traits (GM2, GD2) (**Fig. S11**).

Heterogeneity in individual cerebral organoids

To identify abnormal COs, we sorted them based on three parameters: (i) a sum of all protein marker levels (PML), (ii) a sum of all lipid marker levels (LML), and (iii) the ratio of neuronal protein marker (sum of DCX, TUBB3, MAP2, and NEFM) to the glial marker levels (sum of FABP7 and S100B) (NGR) (**Tab. S9**). We excluded GFAP from the ratio as it was highly abundant in later time points. Parameters were calculated for each CO, and median values were determined at each time point (TP). The acceptance cut-off was +/- 35 % of the median (MED). Values within the 35% upper/lower limit of the median received a score of 0. Values outside the 35% interval received a score of 1 for PML/LML and 2 for the NGR:

$$\text{PML/LML} = 1;\ \text{NGR} = 2 \text{ if}$$
$$\text{MED(x levels at TP)} \pm 0.35*\text{MED(x levels at TP)}$$
$$\text{where x = PML, GM or NGR}$$
$$\text{Total score} = \text{Score(PML)} + \text{Score(LML)} + \text{Score(NGR)}$$

We assigned a higher score to NGR as a critical parameter of cellular differentiation and compositional changes in CO, whereas PML and LML parameters identify fewer specific discrepancies in the differentiation efficiency. For instance, the glia-to-neuron ratio is used to count human brain cells[27]. We summed up each parameter's score, and COs with a total score >2 were removed as outliers (**Tab. S9**). We excluded an abnormal CO at each time point (**Fig. S16 and Tab. S9**), reducing the coefficient of variation among biological replicates considerably (**Tab. S8**).

Major lipid characterization

The lipid extract was analyzed using a 1290 Infinity II UHPLC (Agilent) system coupled with the 6469 Triple Quadrupole mass spectrometer (Agilent). 1 µL of lipid extract was injected on the reverse phase microbore column (CSH, 1 mm *100 mm, 1.7µm, Waters), separated at 100µl/min flow rate over 15 min. For the gradient elution, mobile phase A was 10 mM ammonium formate in acetonitrile: water (60:40), and mobile phase B was 10 mM ammonium formate in Isopropanol: acetonitrile (90:10). The gradient elution program was : 0 min 15 % B, 1.86 min 30% B , 2.32 min 48%, 9.5 min 82% B, 12.5 to 13.5 min 99% B and 13.5 to 15 min column re-equilibration. The positive mode jet stream source parameters were gas temp 200 0C, gas flow 14 l/min, nebulizer pressure 45 psi, sheath gas temp 400 0C, and sheath gas flow 8 l/min capillary voltage 4 kV, nozzle voltage 500v, and unit resolution for Q1 and Q3. Data were acquired in positive mode using the dynamic multiple reaction monitoring (MRM) mode, 2 min retention time window for each transition. Raw data files were processed using Mass Hunter Quantitative analysis (B.07.00, Agilent Technologies) software. Mass spectrometric fragmentation behavior of different lipid classes is well established. For the multiple reaction monitoring (MRM) based on targeted lipid identification1–3, a list

of lipid species was prepared and identified by injecting the QC sample. Our optimized microflow LC-MS/MS method identified 351 lipid species across 25 lipid classes as listed in the table below - Carnitine (Car), Cholesteryl ester (CE), Free Cholesterol (FC), Ceramide (Cer), Dihydroceramide (dhCer), Sphingomyelin (SM), Hexosylceramide (HexCer), Dihexosylceramide (Hex2Cer), Trihexosylceramide (Hex3Cer), Diglycerides (DG), Triglycerides (TG), Lysophosphatidylcholine (LPC), Lysophosphatidylethanolamine (LPE), Phosphatidylcholine (PC), Phosphatidylethanolamine (PE), Phosphatidylserine (PS), Phosphatidylinositol (PI), Phosphatidylglycerol (PG), and Sphingoid bases (Sph).

Lipid classes	No. of species characterized
Car	14
CE	15
FC	1
Cer	26
dhCer	11
SM	30
HexCer	6
Hex2Cer	5
Hex3Cer	3
DG	13
TG	56
LPC	14

Lipid classes	No. of species characterized
LPC-O	3
LPC-P	3
LPE	3
PC	38
PC-O	21
PC-P	14
PE	21
PE-O	14
PS	14
PI	16
PG	7
Sph	3

Supplementary figures:

Figure S1. Localization of neuronal (DCX, TUBB3, MAP2, NEFL, NEFM, SYN1) and astrocytic (S100B, CD44, GFAP) markers and housekeeping proteins (ACTB, GAPDH)

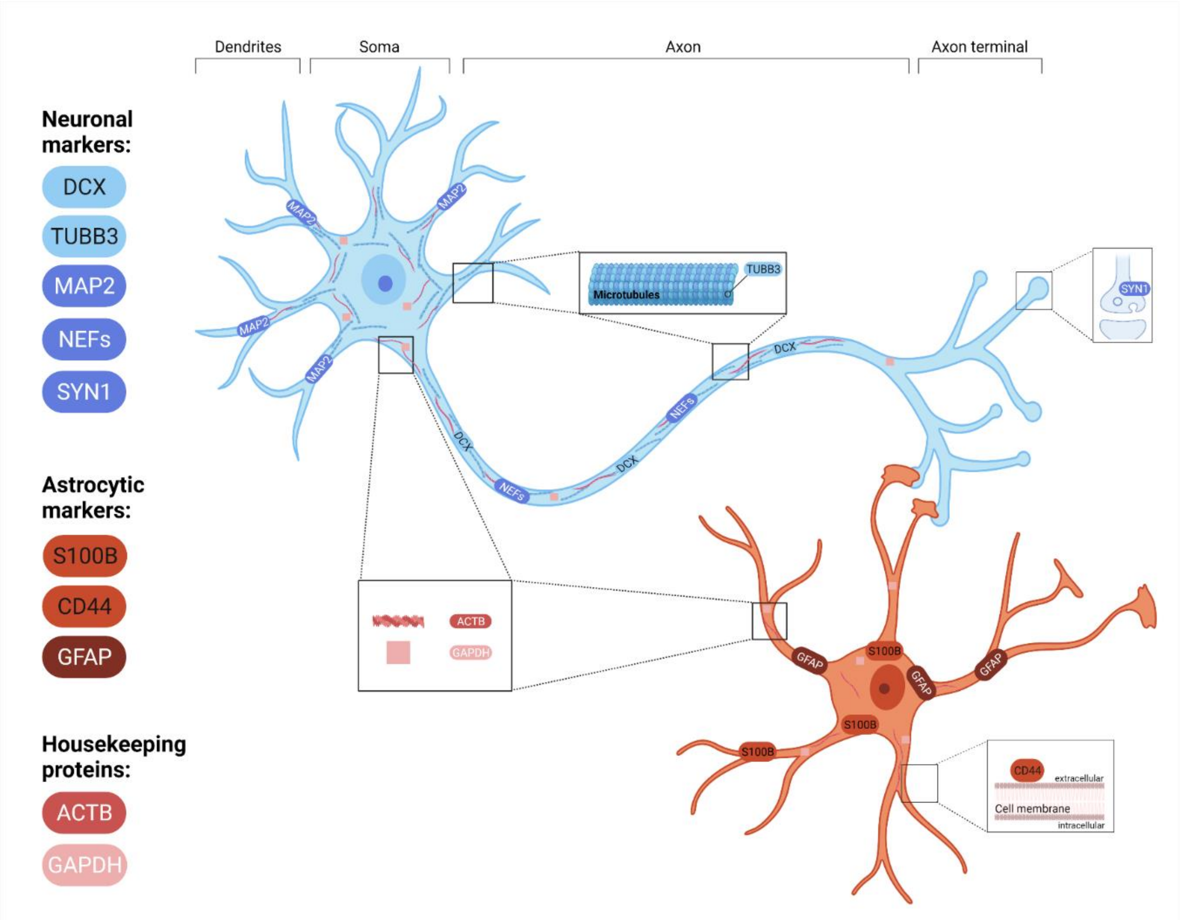

Figure S2. Protein identification and relative quantification workflow. Proteotypic peptides were pre-selected in NeXtProt, prioritizing peptides with experimental data in PeptideAtlas. The top 4-5 transitions were selected to generate the SRM library applied to the QC sample for tentative peptide identification using the RT prediction model. Peptides with expected SRM signature and RT were utilized further for protein assays. Heavy-labeled synthetic peptide internal standards and a scheduled SRM acquisition mode were used for relative protein quantitation. Quantifier transitions were selected based on the low %CV of L/H ratios between technical replicates.

AA = amino acids, QC = quality control pooled sample, RT = retention time, L = light peptide, H = heavy labeled peptide, CV = coefficient of variation

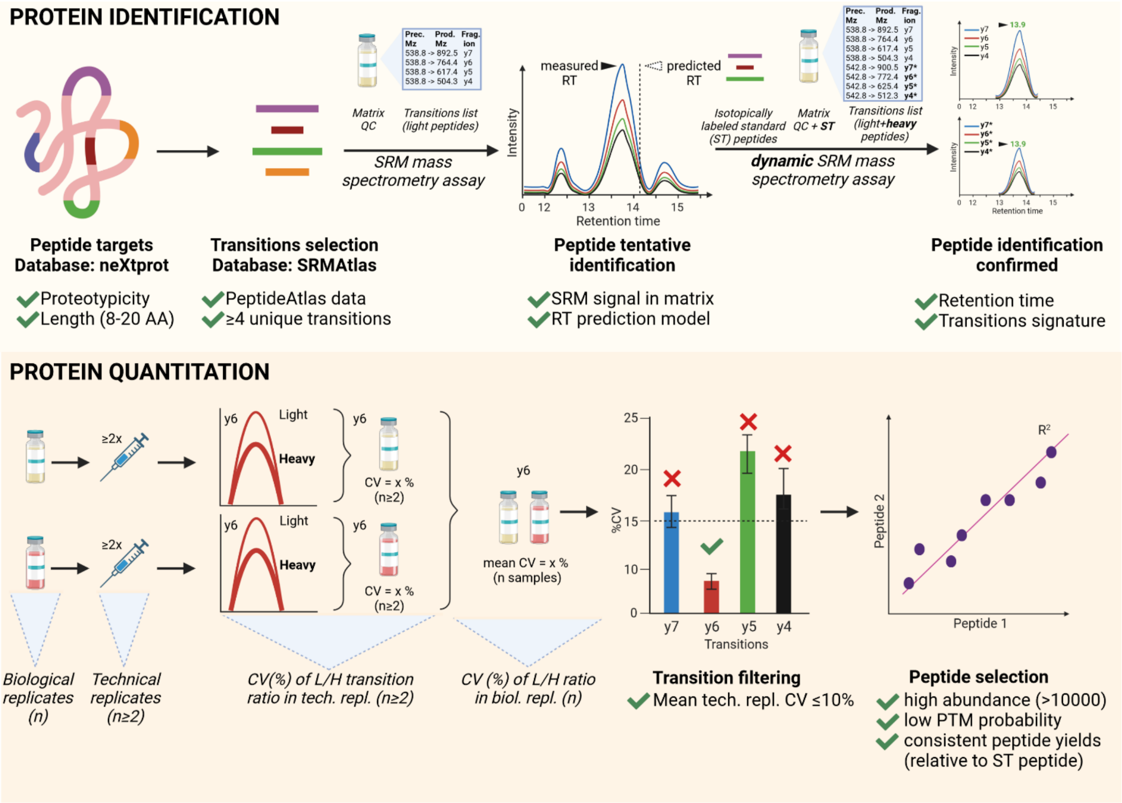

Figure S3. Mass spectra for the 13C labeled GM3 and GM1 synthesized in-house

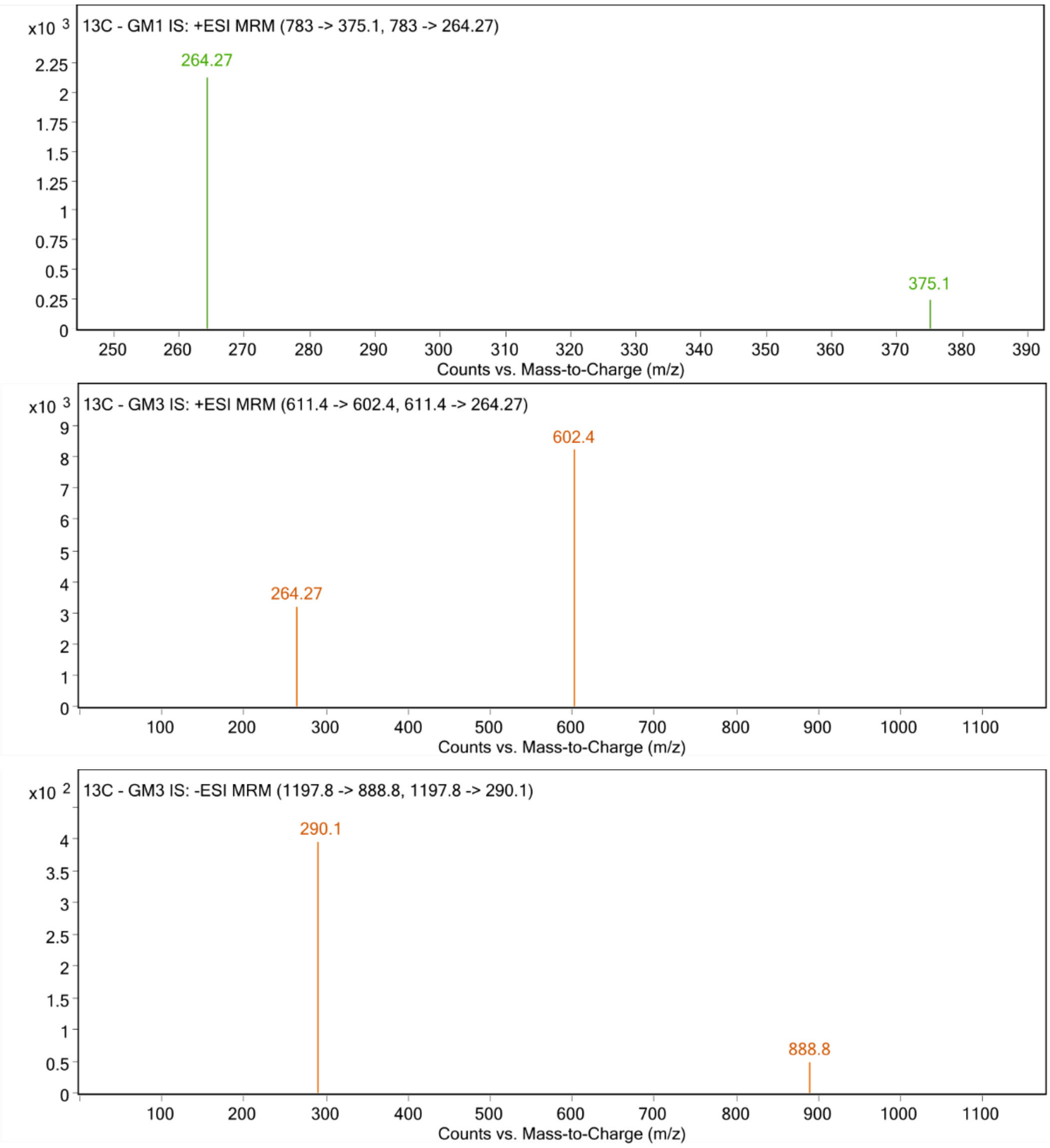

Figure S4. Chromatograms for the (a) gangliosides analysed in the positive and negative mode along with the labeled standards (b) protein analysed with appropriate light peptide underneath the heavy peptide

a

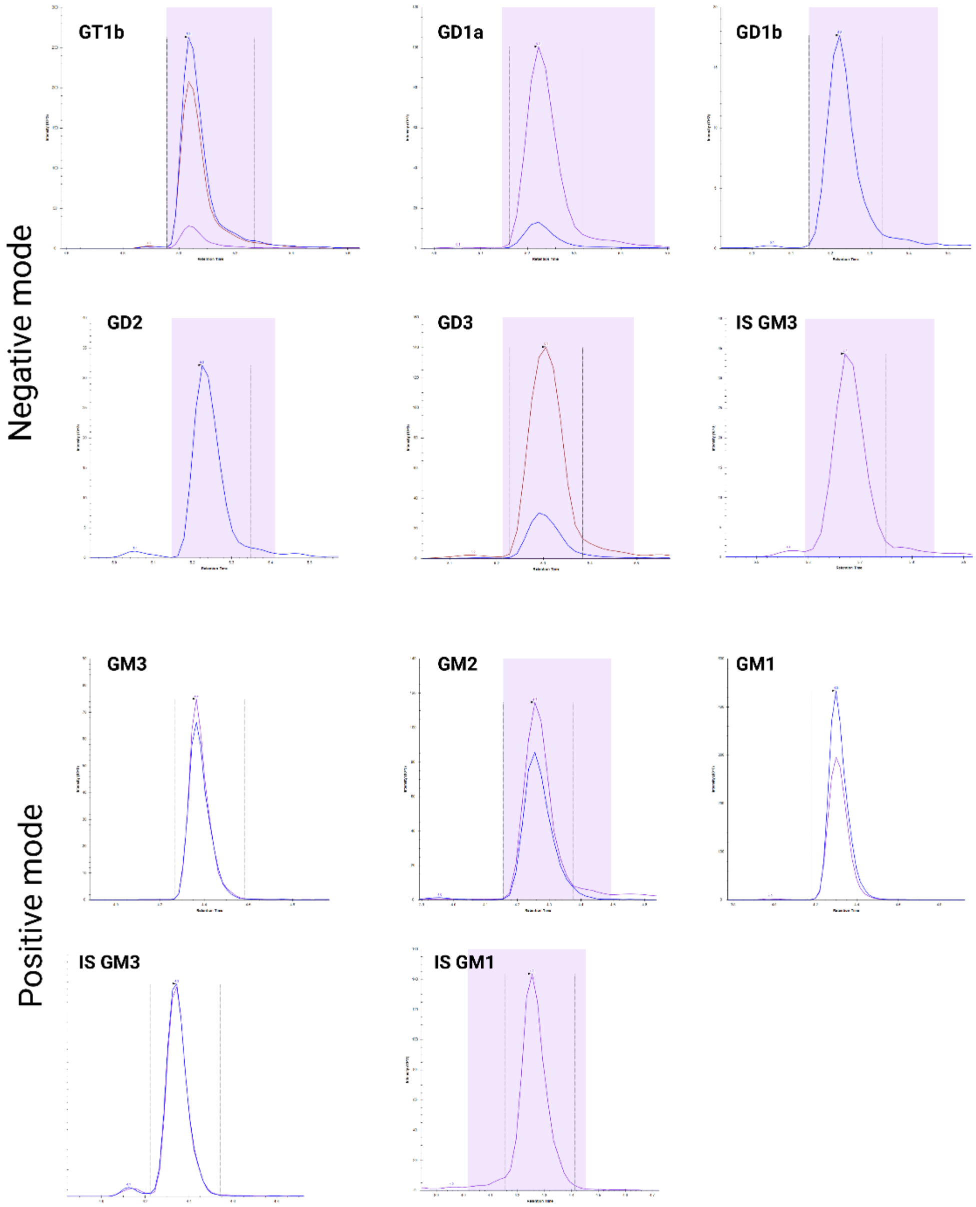

b

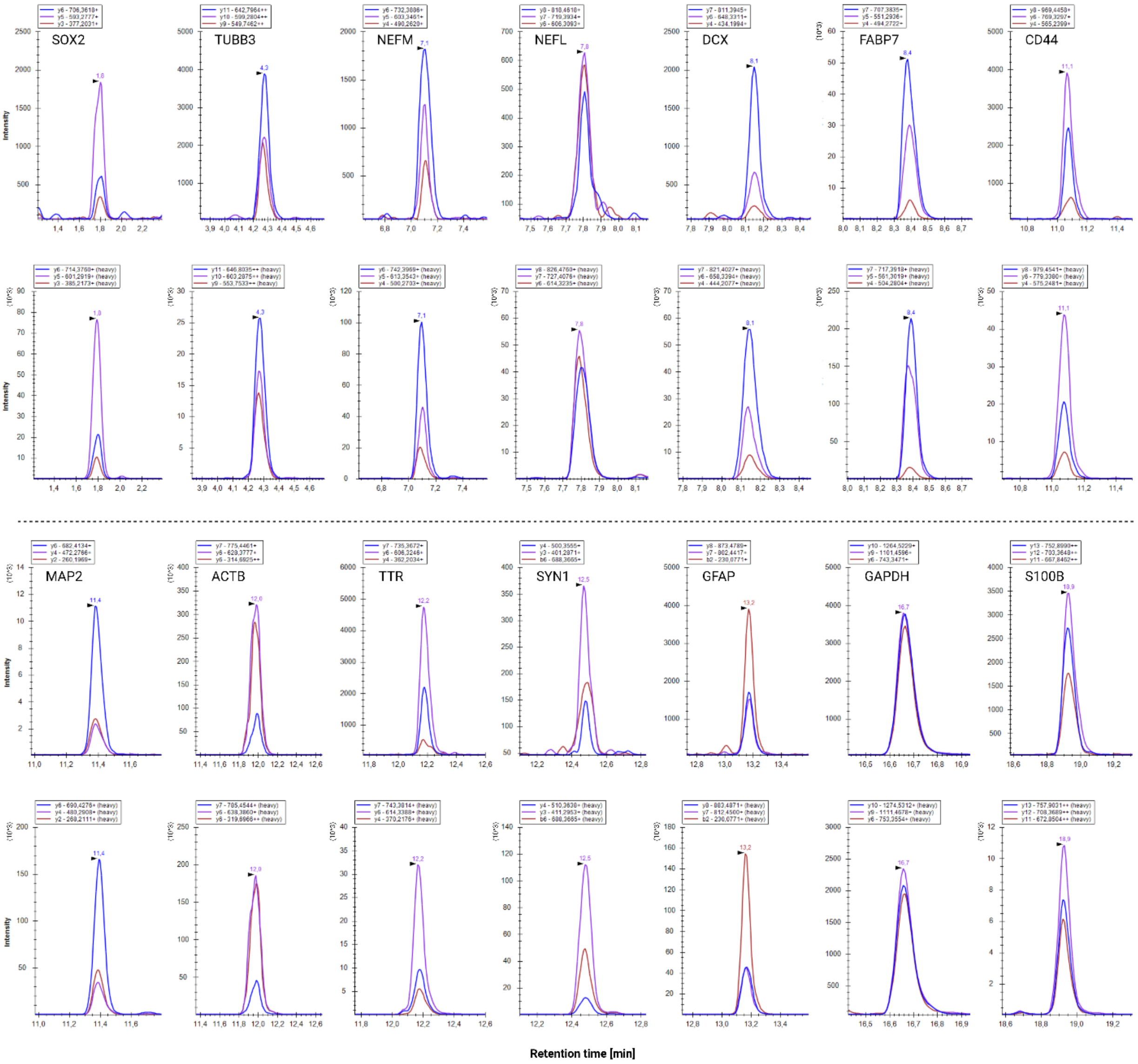
SOX2
TUBB3
NEFM
NEFL
DCX
FABP7
CD44
MAP2
ACTB
TTR
SYN1
GFAP
GAPDH
S100B
Intensity
Retention time [min]

Figure S5. Trypsin digestion efficiency after 2, 4, and 16 hours of incubation

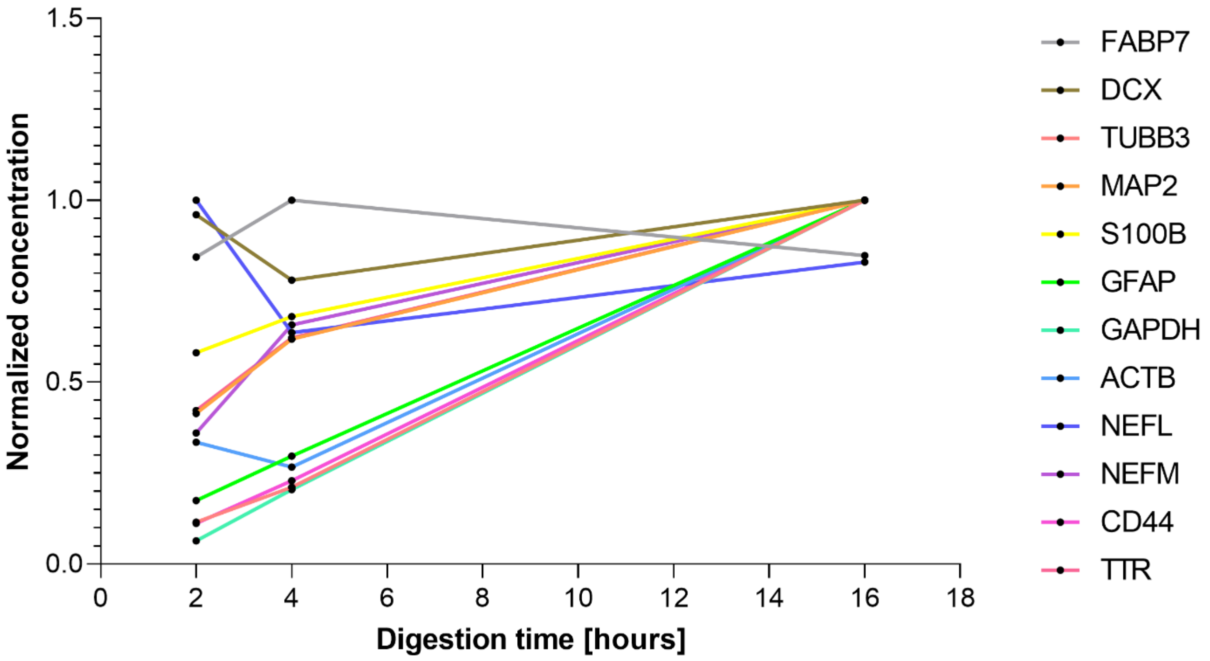

Figure S6. Analytical parameters of proteomic protocol. (a) Solid-phase extraction method (SPE) recovery of standard peptides was on average 87 % (n=2-4). (b) The standard peptide response in the cerebral organoid matrix was, on average, 32% lower relative to the standard peptide response in the neat solvent. (c) The optimal sample injection amount was equivalent to 6 µg of total protein onto the LC column for maximal sensitivity of the protein assay (n=2). On average, the response of synthetic peptide internal standards was reduced by 24 % for the 6 µg total protein equivalent injection, relative to the neat solvent. (d) Normalized concentrations of target proteins at three sample dilution levels, CV between the three calculated concentrations was, on average, 15 %.

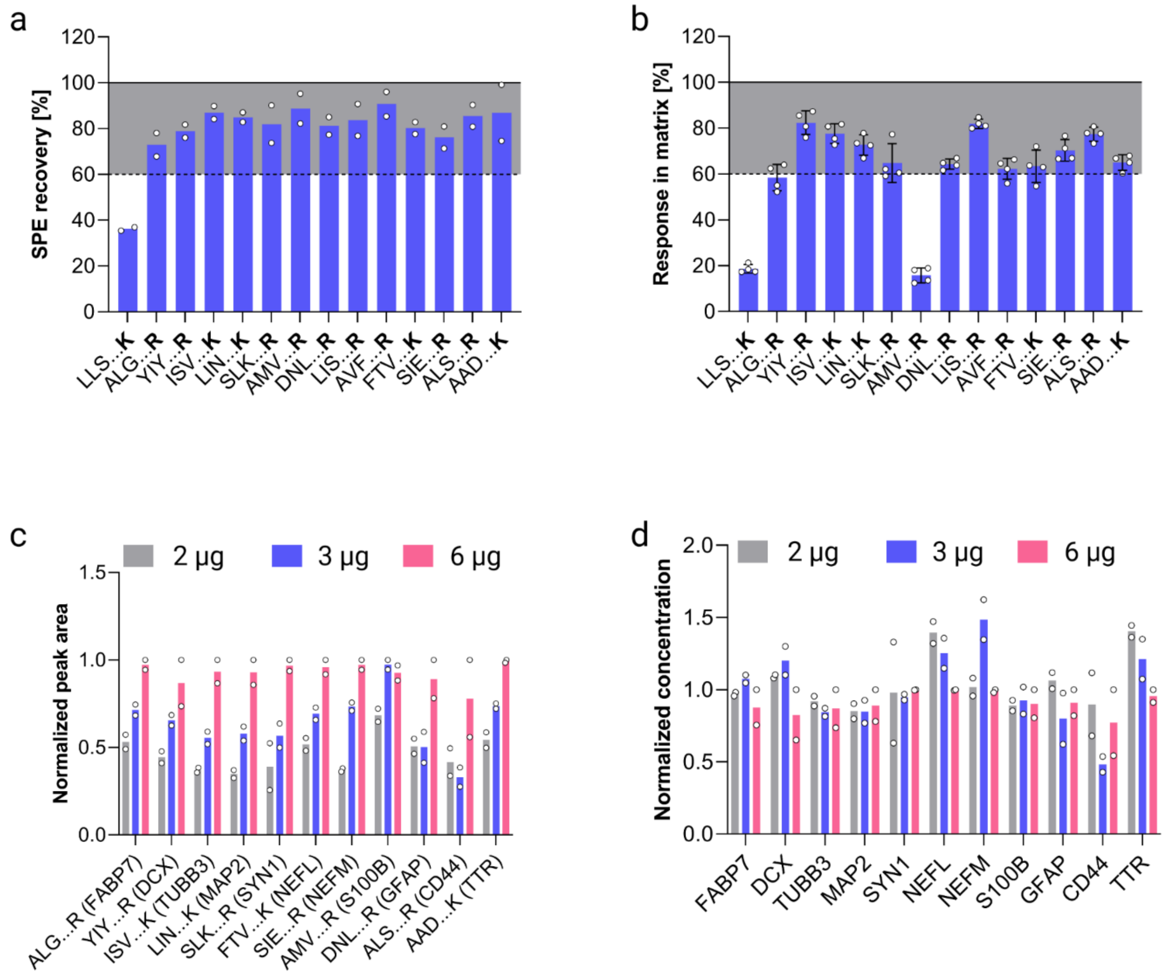

Figure S7. Calibration curves for ganglioside isotopically-labeled standards

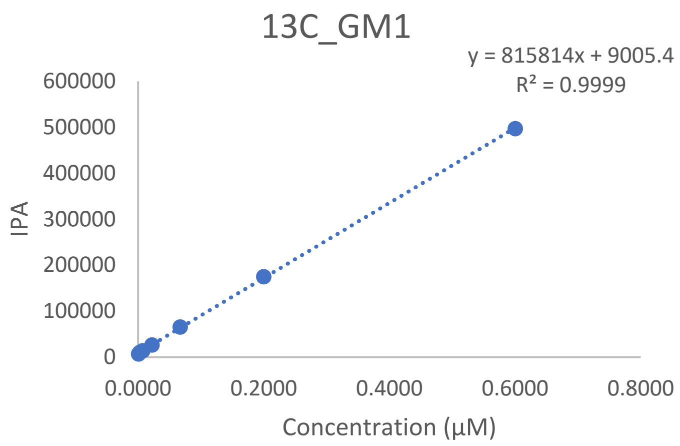

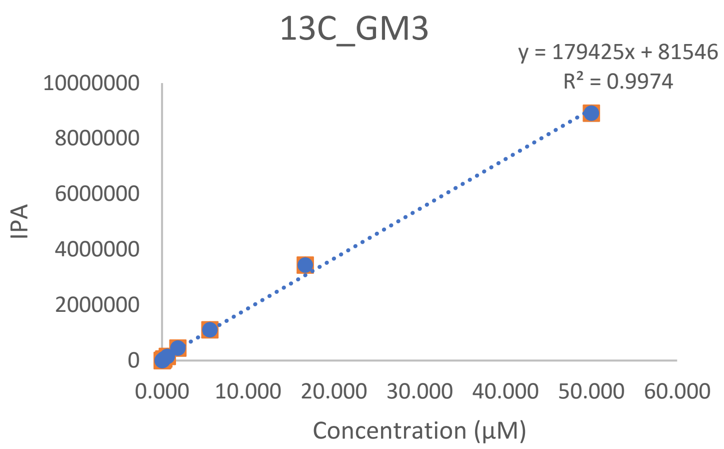

Figure S8. Calibration curves of synthetic isotopically labeled peptide internal standards used for protein assays

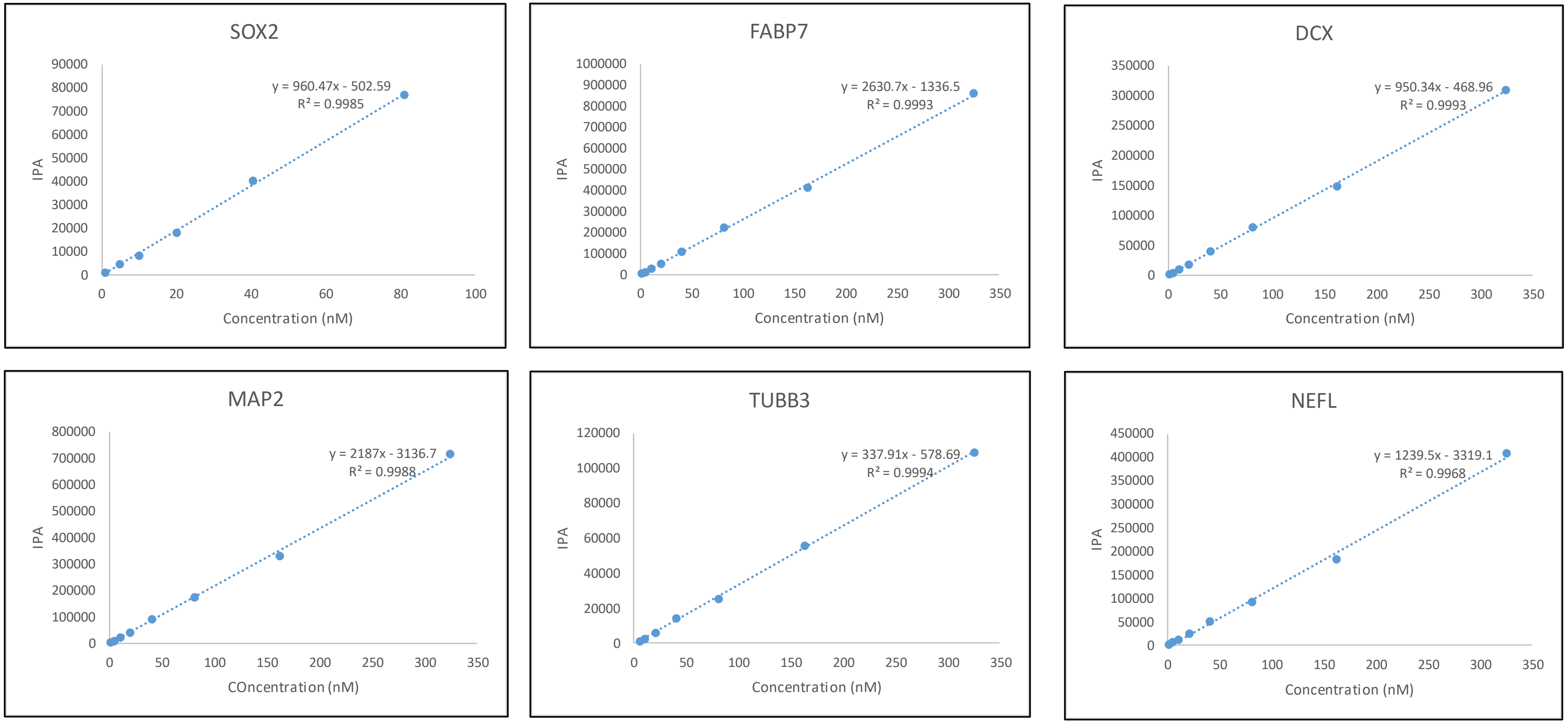

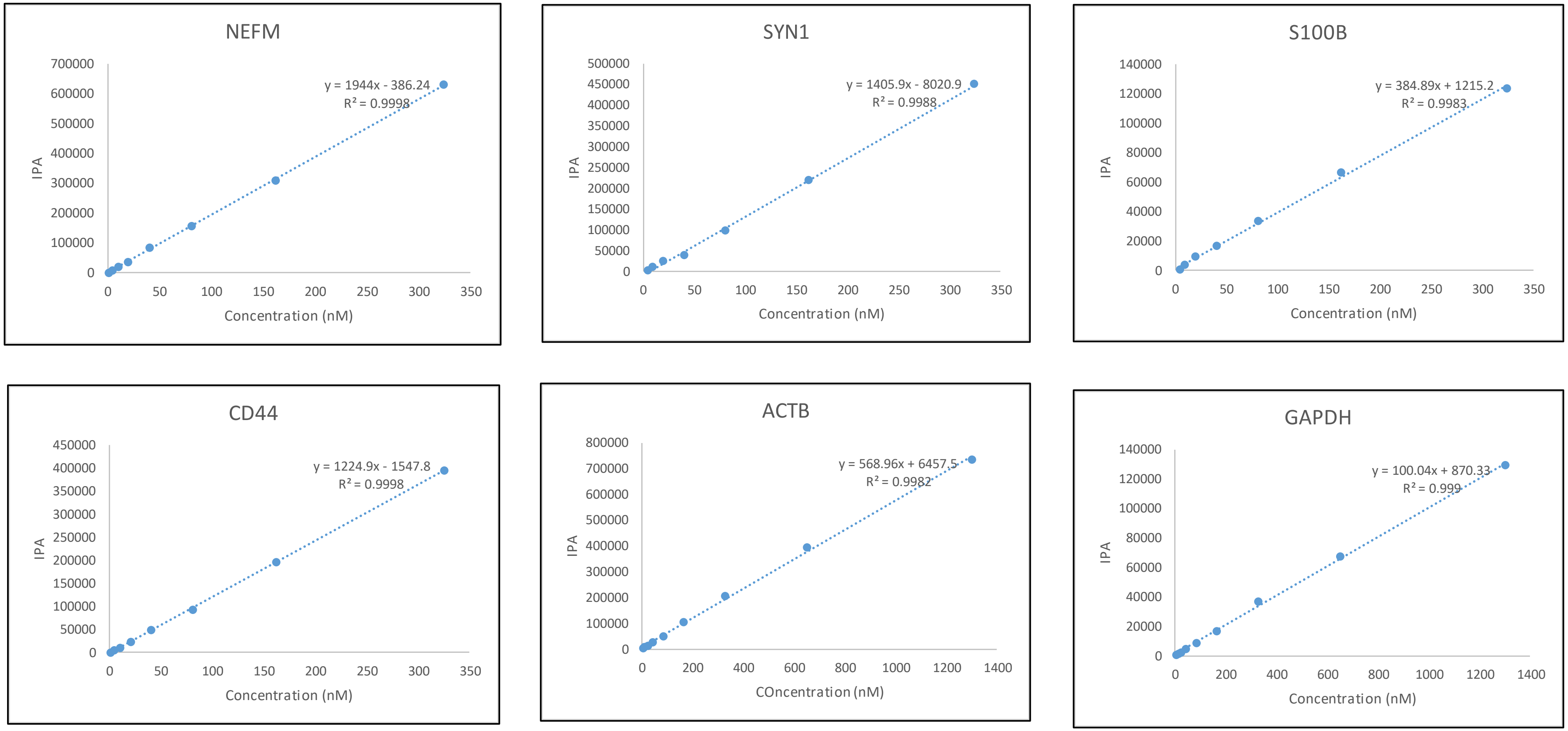
NEFM
y = 1944x - 386.24
R² = 0.9998
IPA
Concentration (nM)
SYN1
y = 1405.9x - 8020.9
R² = 0.9988
IPA
Concentration (nM)
S100B
y = 384.89x + 1215.2
R² = 0.9983
IPA
Concentration (nM)
CD44
y = 1224.9x - 1547.8
R² = 0.9998
IPA
Concentration (nM)
ACTB
y = 568.96x + 6457.5
R² = 0.9982
IPA
COncentration (nM)
GAPDH
y = 100.04x + 870.33
R² = 0.999
IPA
Concentration (nM)

Figure S9. Cell recovery solution (CRS) treatment to remove Geltrex sample matrix

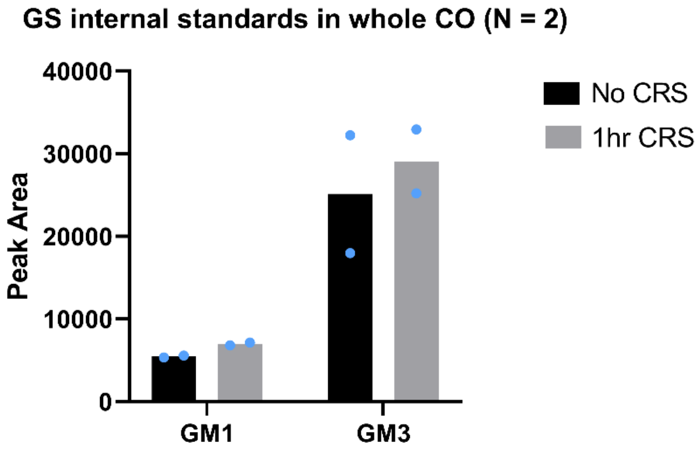

Figure S10. Representative chromatograms of a peptide and a ganglioside in blank, QC, CO D48, and CO D160 samples.

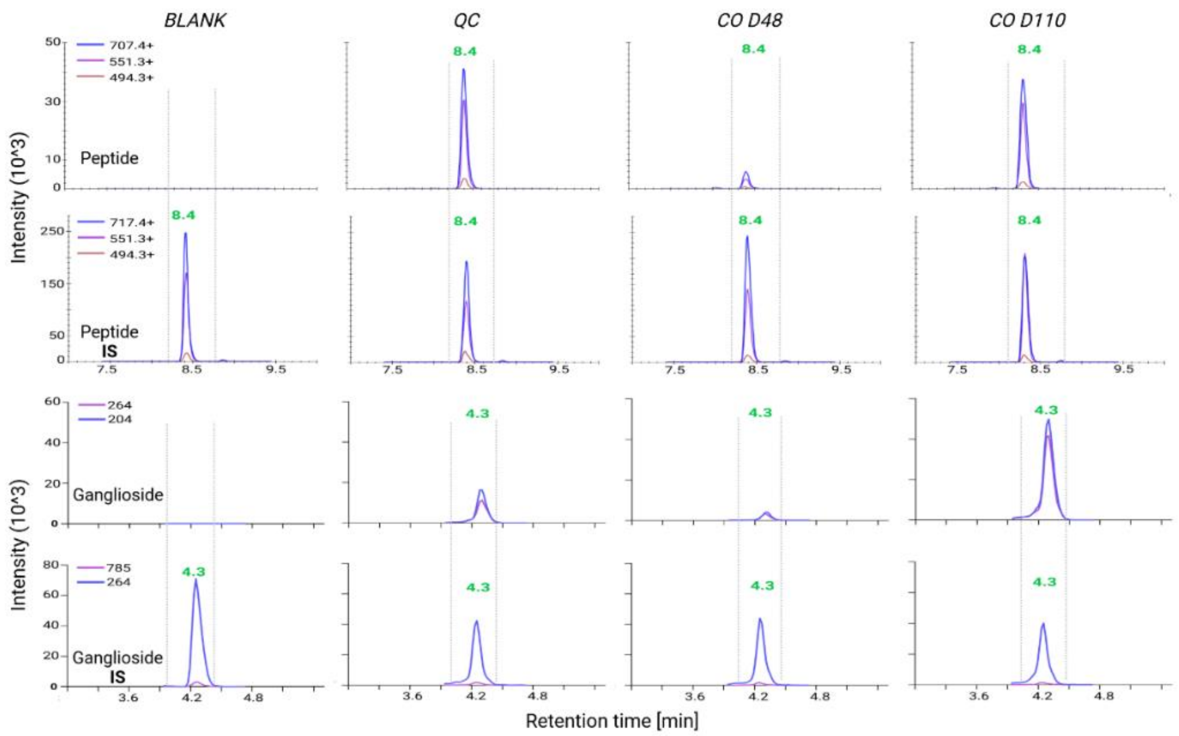

Figure S11. The time-trends of NSCs markers (SOX2, GD3); neuron-specific markers (DCX, NEFL, TUBB3, MAP2, SYN1, GD1a, GD1b, and GT1b); RGCs marker (FABP7); and astrocyte-specific markers (S100B, GFAP, and GM2). Concentrations <LOD are highlighted in red (•), >LOD and <LOQ in grey (•), zero values with empty points (o). Protein marker and GS levels were normalized to house-keeping protein ACTB.

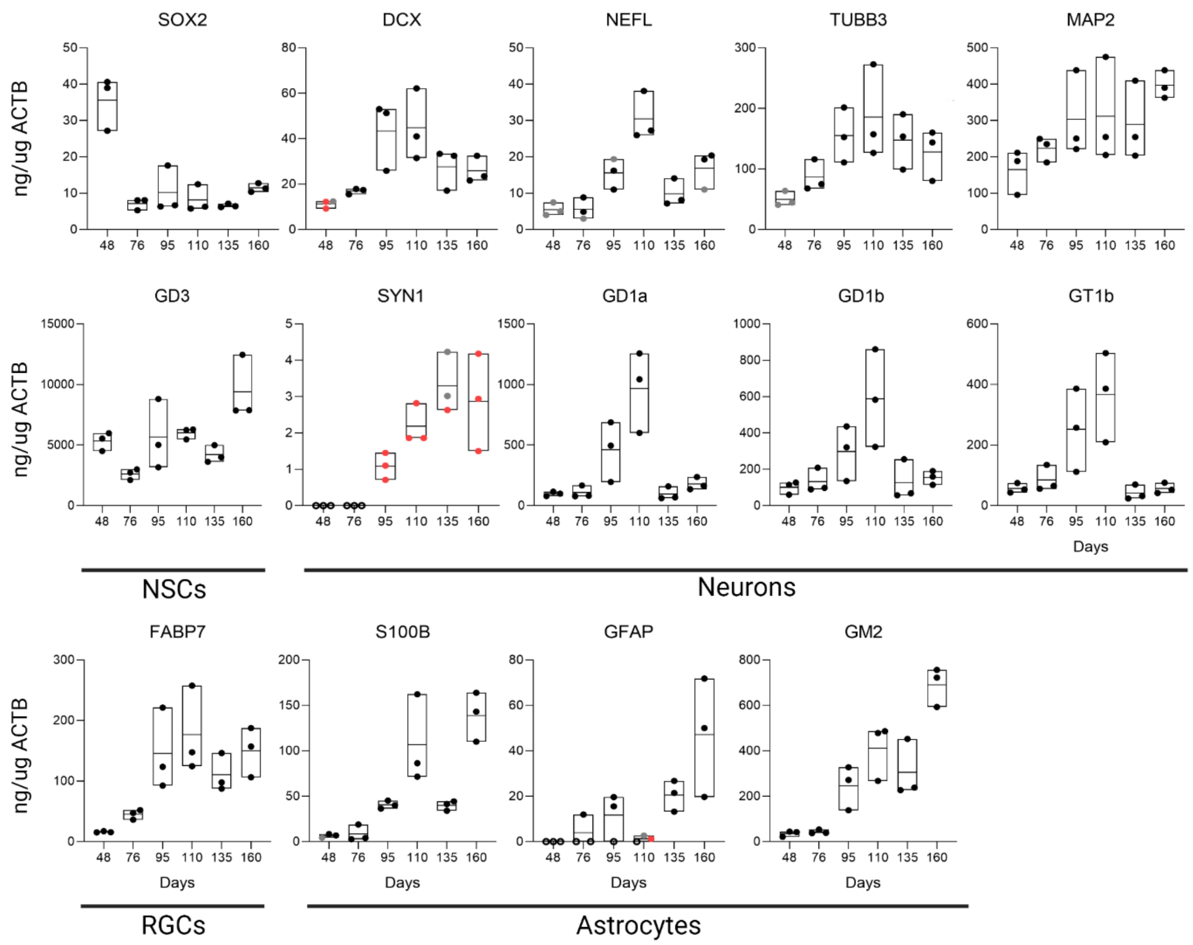

Figure S12. Housekeeping proteins ACTB and GAPDH represent the total cell mass during cerebral organoid proliferation

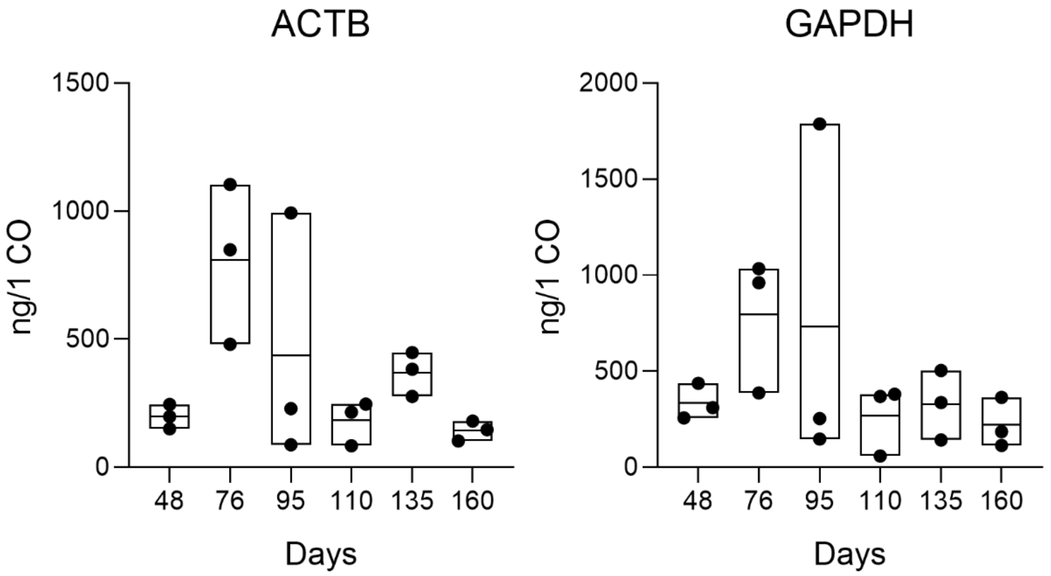

Figure S13. Temporal trends of selected protein markers comparing reference methods (i.e., WB, qPCR) with MS assays

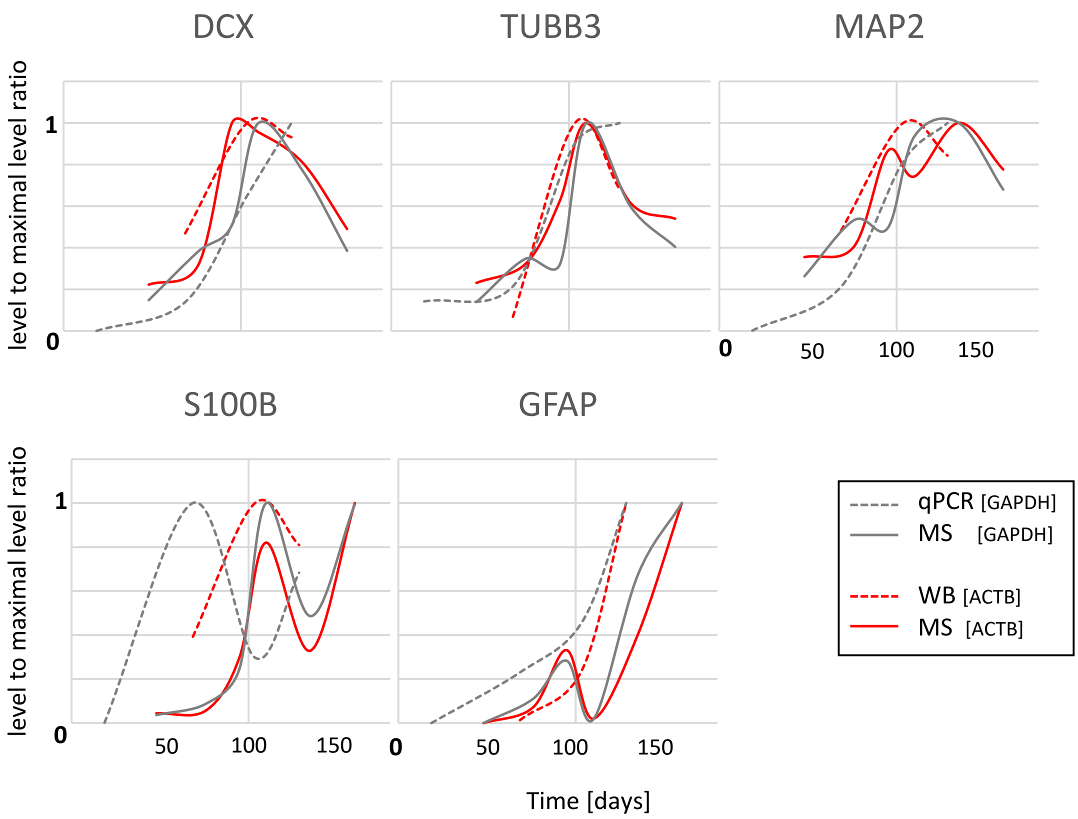

Figure S14. The time-trends of major lipids as observed in the cerebral organoids. The box plots are the sum of all lipid-species from the same class. The peak area sum has been normalized to to house-keeping protein ACTB.

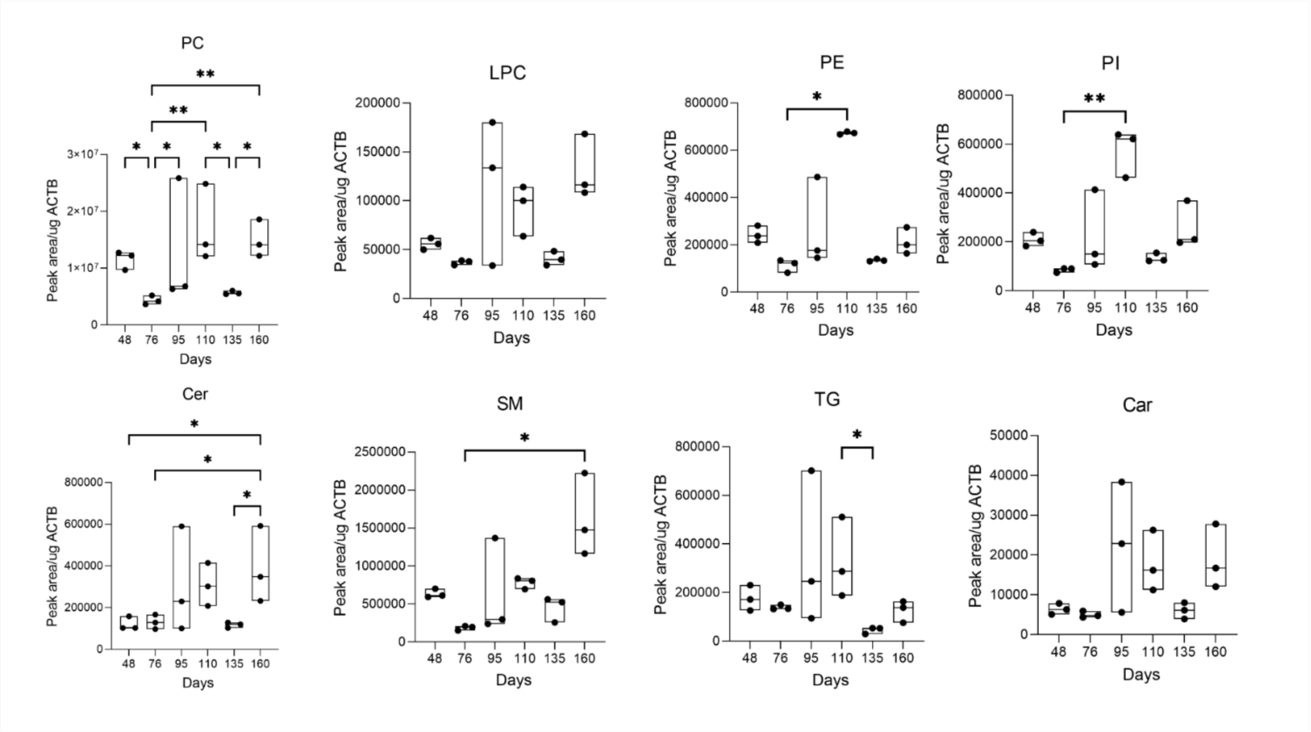

Figure S15. The cluster analysis of proteins and gangliosides during early and late neurogenesis in cerebral organoids. **(a)** The early neurogenesis (D48, D76, D95; n=9) is represented by principal clusters (Ia, Ib, II). Cluster Ia. associated neuronal and astrocytic gangliosides, neuronal protein traits (except for MAP2), and RGCs markers. Cluster Ib. associated astrocytic protein markers and MAP2. Cluster II. grouped peripheral gangliosides and NSCs protein markers (SOX2, GM3, GD3). **(b)** Five distinct clusters (IIIa; IIIb1; IIIb2; IVa; IVb) represent the late neurogenesis (D110, D135, D160; n=9). Cluster IIIa associates immature neuronal markers, cluster IIIb1 associates GM3 and TTR, and cluster IIIb2 associates mature neuronal markers with an RGCs marker (FABP7). Cluster IVa associates mature astrocytic markers GFAP and CD44 and cluster IVb astrocytic S100B, MAP2, SOX2, and astrocytic gangliosides (i.e., GM2, GD2).

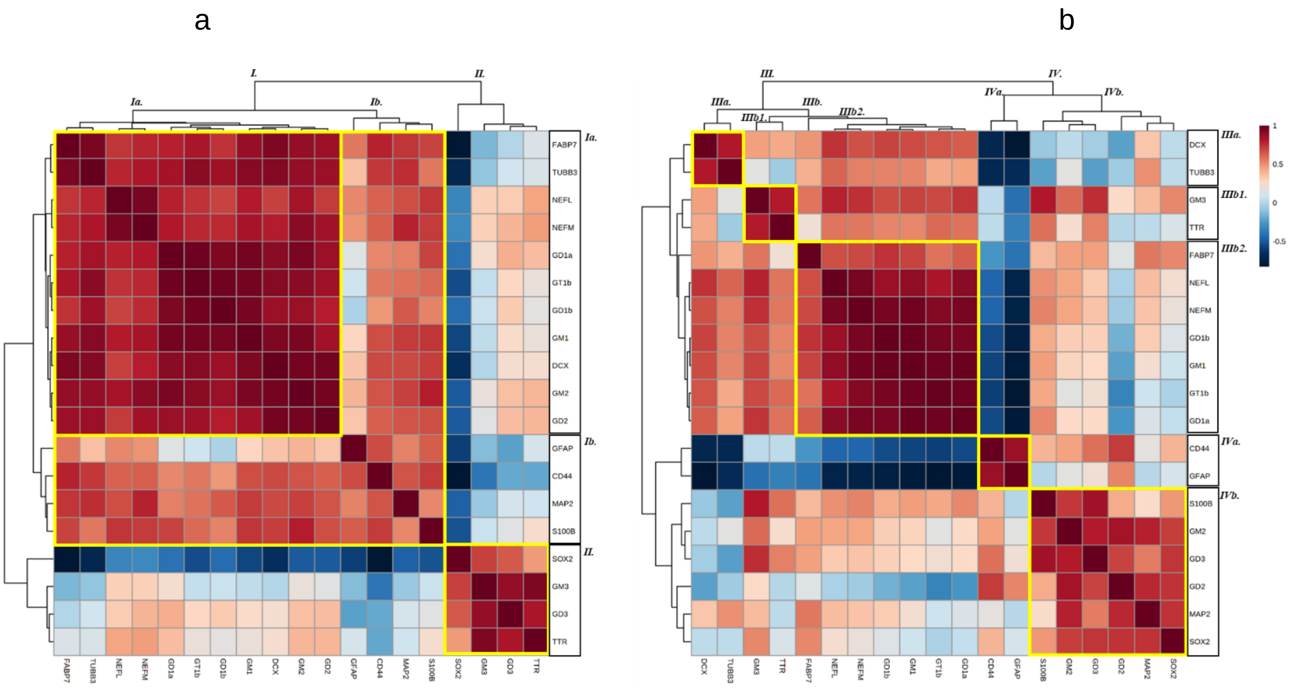

Figure S16. Variability scores the sum of lipid markers levels, the sum of protein markers levels, and neurons to glia ratio of individually profiled cerebral organoids as listed in Table S9

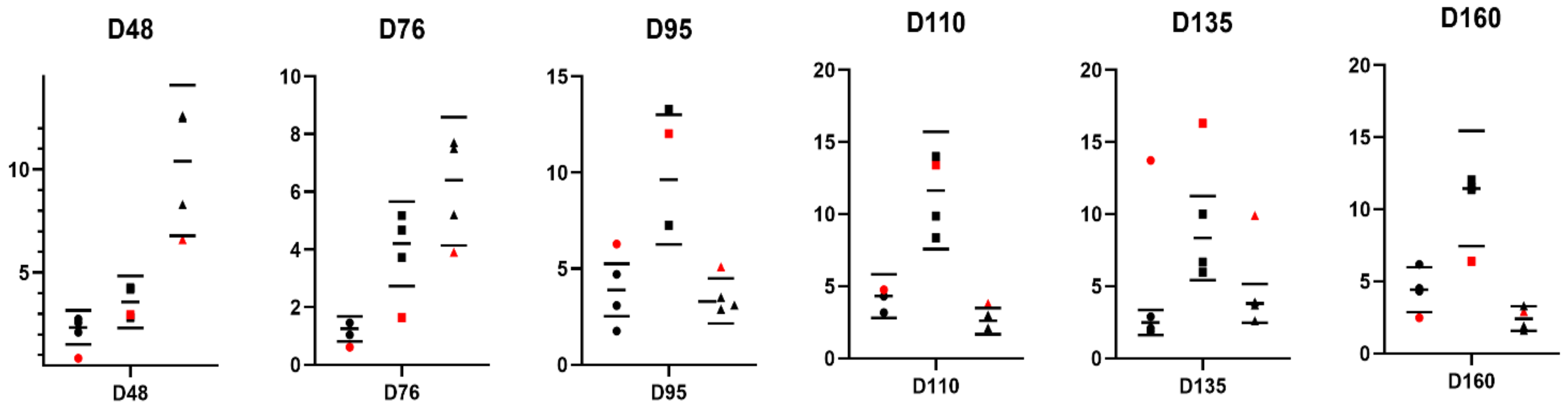

Supplementary tables

Table S1. Protein markers profiled in cerebral organoids by mass spectrometry. Immuno-based techniques and qPCR were used as a reference method for some protein markers

Gene	Protein	Neural cell type specificity	Subcellular localization	Molecular function	Wblot	qPCR	Immuno
DCX	Neuronal migration protein doublecortin	neuron (immature)	neuron projection, cytoskeleton, axoneme	microtubule-binding, neuronal dispersion	✓	✓	✓
TUBB3	Tubulin beta-3 chain	neuron (immature)	axon, cytoskeleton, microtubule	structural constituent of cytoskeleton	✓	✓	✓
MAP2	Microtubule-associated protein 2	neuron	dendrite, cytoskeleton, microtubule	microtubule-binding, tau binding	✓	✓	✓
NEFL	Neurofilament light polypeptide	neuron	axon, cytoskeleton, neurofilament	structural constituent of cytoskeleton	✓	n. a.	✓
NEFM	Neurofilament medium polypeptide	neuron	axon, cytoskeleton, neurofilament	structural constituent of cytoskeleton	n. a.	n. a.	n. a.
SYN1	Synapsin-1	neuron	axon, dendrite, cytoskeleton, synaptic vesicle	transporter activity binds to the cytoskeleton	✓	n. a.	✓
CD44	Cell-surface glycoprotein	astrocyte	cell projection, plasma membrane	transmembrane receptor, collagen-binding	n. a.	n. a.	n. a.
GFAP	Glial fibrillary acidic protein	radial glial cell/glial cell/astrocyte	cytoskeleton	structural constituent of cytoskeleton	✓	✓	✓
S100B	Calcium-binding protein B	radial glial cell/glial cell/astrocyte	nucleus, cytoplasm	calcium-dependent regulation, tau binding	✓	✓	n. a.
SOX2	SRY-box transcription factor	neural stem cell/radial glial cell	Nucleus	neuronal differentiation suppression	✓	✓	✓
FABP7	Fatty acid-binding protein	radial glial cell/glial cell	Cytoplasm	radial glial fiber system, immature neurons migration	n. a.	n. a.	n. a.
TTR	Transthyretin	epithelial cell (Choroid plexus)	cytoplasm, secreted	thyroxine hormone transport from the bloodstream	n. a.	✓	n. a.
ACTB	Beta-actin	all neural cells	Cytoplasm	structural constituent of the cytoskeleton, tau binding	✓	n. a.	n. a.
GAPDH	Glyceraldehyde 3-P dehydrogenase	all neural cells	Cytoplasm	key enzyme in glycolysis	n. a.	✓	n. a.

✓ = detected; n. a. = not analyzed

Table S2. Selected reaction monitoring (SRM) library of precursor/product ion transitions for the analysis of gangliosides in positive and negative ion detection modes

Ganglioside	Precursor M/z	Product M/z	Collision Energy (eV)	Retention Time (min)	Adduct
GM1	773.9	366.1*	9	4.3	M+2H
GM1	773.9	264.3	9	4.3	M+2H
13C-GM1 IS	783.0	375.1*	41	4.3	M+2H
13C-GM1 IS	783.0	264.3	41	4.3	M+2H
GM2	703.9	695.0	9	4.3	M+H+Na
GM2	703.9	264.3*	9	4.3	M+H+Na
13C-GM3 IS	611.4	602.4	9	4.35	M+H+Na
13C-GM3 IS	611.4	264.3	9	4.35	M+H+Na
GM3	602.38	593.4	9	4.35	M+H+Na
GM3	602.38	264.3	9	4.35	M+H+Na
GM3	1179.7	888.8	37.6	5.7	M-H
GM3	1179.7	290.1*	37.6	5.7	M-H
13C-GM3 IS	1197.8	888.8	38.1	5.7	M-H
13C-GM3 IS	1197.8	290.1*	38.1	5.7	M-H
GD2	836.5	290.1*	26.9	5.2	M-2H
GD3	734.9	581.1	23.8	5.3	M-2H
GD3	734.9	392.9	23.8	5.3	M-2H
GD3	734.9	290.1*	23.8	5.3	M-2H
GD1a/b	917.5	888.6	29.4	5.2	M-2H
GD1a/b	917.5	290.1*	29.4	5.2	M-2H
GD1b	917.5	581.2*	29.4	5.2	M-2H
GT1b	1063.0	917.6	34	5.0	M-2H
GT1b	1063.0	581.1	34	5.0	M-2H
GT1b	1063.0	290.1*	34	5.0	M-2H

*Transitions used for the quantitation of the respective GSs.

Table S3. Synthetic isotopically labeled peptides for protein assays: signal reproducibility, recovery, and matrix effects

**Protein [gene name]**	**Quantifier ST peptide**	**signal stability** in solvent **CV [%]**	**signal stability** in matrix **CV [%]**	**SPE recovery** in matrix **[%]**	**matrix effect** in matrix **[%]**
SOX2	LLSETEK	7	9	42	79
FABP7	ALGVGFATR	15	8	80	27
DCX	YIYTIDGSR	12	9	84	14
TUBB3	ISVYYNEASSHK	6	11	91	21
MAP2	LINQPLPDLK	14	8	91	21
NEFL	FTVLTESAAK	18	10	91	31
NEFM	SIELESVR	14	8	83	23
S100B	AMVALIDVFHQYSGR	11	6	107	83
GFAP	DNLAQDLATVR	12	5	85	30
CD44	ALSIGFETCR	16	8	89	17
TTR	AADDTWEPFASGK	16	9	91	17
ACTB	AVFPSIVGR	11	10	97	34
GAPDH	LISWYDNEFGYSNR	14	5	87	15
SYN1	SLKPDFVLIR	9	8	98	29
**AVERAGE [%]**		**13**	**8**	**87**	**32**

*matrix effect was determined by the ratio of recovery of internal standards signal in a neat solvent to the recovery of internal standard in the sample matrix.

Table S4. SRM library for synthetic isotopically labeled peptides for protein assays. Quantifier SRM transitions marked in bold.

Protein number	Gene name	Peptide sequence	Precursor Adduct	Precursor Mz	Product Adduct	Product Mz	Fragment Ion	Collision Energy [eV]	Retention Time [min]
P48431	*SOX2*	LLSETEK	[M+2H]	410.2	[M+H]	706.4	y6	13.7	1.8
			[M+2H]	410.2	[M+H]	593.3	y5	13.7	1.8
			[M+2H]	410.2	[M+H]	377.2	y3	13.7	1.8
		LLSETE**K**	[M+2H]	414.2	[M+H]	714.4	y6	13.7	1.8
			**[M+2H]**	**414.2**	**[M+H]**	**601.3**	**y5**	**13.7**	**1.8**
			[M+2H]	414.2	[M+H]	385.2	y3	13.7	1.8
		DMISMYLPGAEVPEPAAPSR	[M+3H]	711.0	[M+H]	824.4	y8	20.8	20.2
			[M+3H]	711.0	[M+H]	598.3	y6	20.8	20.2
			[M+3H]	711.0	[M+2H]	639.3	y13	20.8	20.2
		DMISMYLPGAEVPEPAAPS**R**	[M+3H]	714.3	[M+H]	834.4	y8	20.8	20.2
			[M+3H]	714.3	[M+H]	608.3	y6	20.8	20.2
			[M+3H]	714.3	[M+2H]	644.3	y13	20.8	20.2
O15540	*FABP7*	QVGNVTKPTVIISQEGDK	[M+3H]	638.3	[M+H]	776.4	y7	18.2	7.7
			[M+3H]	638.3	[M+H]	663.3	y6	18.2	7.7
			[M+3H]	638.3	[M+2H]	843.5	y16	18.2	7.7
		QVGNVTKPTVIISQEGD**K**	[M+3H]	641.0	[M+H]	784.4	y7	18.2	7.7
			[M+3H]	641.0	[M+H]	671.3	y6	18.2	7.7
			[M+3H]	641.0	[M+2H]	847.5	y16	18.2	7.7
		ALGVGFATR	[M+2H]	446.3	[M+H]	707.4	y7	14.8	8.4
			[M+2H]	446.3	[M+H]	551.3	y5	14.8	8.4
			[M+2H]	446.3	[M+H]	494.3	y4	14.8	8.4
		ALGVGFAT**R**	**[M+2H]**	**451.3**	**[M+H]**	**717.4**	**y7**	**14.8**	**8.4**
			[M+2H]	451.3	[M+H]	561.3	y5	14.8	8.4
			[M+2H]	451.3	[M+H]	504.3	y4	14.8	8.4
		SVVSLDGDK	[M+2H]	460.2	[M+H]	733.4	y7	15.3	4.8
			[M+2H]	460.2	[M+H]	634.3	y6	15.3	4.8

			[M+2H]	460.2	[M+H]	547.3	y5	15.3	4.8
		SVVSLDGD**K**	[M+2H]	464.2	[M+H]	741.4	y7	15.3	4.8
			[M+2H]	464.2	[M+H]	642.3	y6	15.3	4.8
			[M+2H]	464.2	[M+H]	555.3	y5	15.3	4.8
O43602	*DCX*	SLSDNINLPQGVR	[M+2H]	706.9	[M+H]	783.4	y7	22.9	11.8
			[M+2H]	706.9	[M+H]	669.4	y6	22.9	11.8
			[M+2H]	706.9	[M+H]	556.3	y5	22.9	11.8
		SLSDNINLPQGV**R**	[M+2H]	711.9	[M+H]	793.5	y7	22.9	11.8
			[M+2H]	711.9	[M+H]	679.4	y6	22.9	11.8
			[M+2H]	711.9	[M+H]	566.3	y5	22.9	11.8
		GIVYAVSSDR	[M+2H]	533.8	[M+H]	797.4	y7	17.5	6.3
			[M+2H]	533.8	[M+H]	634.3	y6	17.5	6.3
			[M+2H]	533.8	[M+H]	563.3	y5	17.5	6.3
		GIVYAVSSD**R**	[M+2H]	538.8	[M+H]	807.4	y7	17.5	6.3
			[M+2H]	538.8	[M+H]	644.3	y6	17.5	6.3
			[M+2H]	538.8	[M+H]	573.3	y5	17.5	6.3
		YIYTIDGSR	[M+2H]	544.3	[M+H]	811.4	y7	17.9	8.2
			[M+2H]	544.3	[M+H]	648.3	y6	17.9	8.2
			[M+2H]	544.3	[M+H]	434.2	y4	17.9	8.2
		YIYTIDGS**R**	**[M+2H]**	**549.3**	**[M+H]**	**821.4**	**y7**	**17.9**	**8.2**
			[M+2H]	549.3	[M+H]	658.3	y6	17.9	8.2
			[M+2H]	549.3	[M+H]	444.2	y4	17.9	8.2
Q13509	*TUBB3*	LHFFMPGFAPLTAR	[M+3H]	535.6	[M+H]	628.4	y6	14.5	19.3
			[M+3H]	535.6	[M+H]	557.3	y5	14.5	19.3
			[M+3H]	535.6	[M+2H]	279.2	y5	14.5	19.3
		LHFFMPGFAPLTA**R**	[M+3H]	539.0	[M+H]	638.4	y6	14.5	19.3
			[M+3H]	539.0	[M+H]	567.3	y5	14.5	19.3
			[M+3H]	539.0	[M+2H]	284.2	y5	14.5	19.3
		ISVYYNEASSHK	[M+3H]	466.6	[M+2H]	642.8	y11	12	4.3
			[M+3H]	466.6	[M+2H]	599.3	y10	12	4.3
			[M+3H]	466.6	[M+2H]	549.7	y9	12	4.3

		ISVYYNEASSH**K**	**[M+3H]**	**469.2**	**[M+2H]**	**646.8**	**y11**	**12**	**4.3**
			[M+3H]	469.2	[M+2H]	603.3	y10	12	4.3
			[M+3H]	469.2	[M+2H]	553.8	y9	12	4.3
P07196	*NEFL*	FTVLTESAAK	[M+2H]	533.8	[M+H]	818.5	y8	17.5	7.8
			[M+2H]	533.8	[M+H]	719.4	y7	17.5	7.8
			[M+2H]	533.8	[M+H]	606.3	y6	17.5	7.8
		FTVLTESAA**K**	[M+2H]	537.8	[M+H]	826.5	y8	17.5	7.8
			**[M+2H]**	**537.8**	**[M+H]**	**727.4**	**y7**	**17.5**	**7.8**
			[M+2H]	537.8	[M+H]	614.3	y6	17.5	7.8
		ALYEQEIR	[M+2H]	511.3	[M+H]	837.4	y6	16.8	6.4
			[M+2H]	511.3	[M+H]	674.3	y5	16.8	6.4
			[M+2H]	511.3	[M+H]	545.3	y4	16.8	6.4
		ALYEQEI**R**	[M+2H]	516.3	[M+H]	847.4	y6	16.8	6.4
			[M+2H]	516.3	[M+H]	684.4	y5	16.8	6.4
			[M+2H]	516.3	[M+H]	555.3	y4	16.8	6.4
P07197	*NEFM*	QASHAQLGDAYDQEIR	[M+3H]	601.3	[M+H]	823.4	y6	16.8	6.5
			[M+3H]	601.3	[M+H]	660.3	y5	16.8	6.5
			[M+3H]	601.3	[M+H]	545.3	y4	16.8	6.5
		QASHAQLGDAYDQEI**R**	[M+3H]	604.6	[M+H]	833.4	y6	16.8	6.5
			[M+3H]	604.6	[M+H]	670.3	y5	16.8	6.5
			[M+3H]	604.6	[M+H]	555.3	y4	16.8	6.5
		SIELESVR	[M+2H]	466.8	[M+H]	732.4	y6	15.5	7.1
			[M+2H]	466.8	[M+H]	603.3	y5	15.5	7.1
			[M+2H]	466.8	[M+H]	490.3	y4	15.5	7.1
		SIELESV**R**	**[M+2H]**	**471.8**	**[M+H]**	**742.4**	**y6**	**15.5**	**7.1**
			[M+2H]	471.8	[M+H]	613.4	y5	15.5	7.1
			[M+2H]	471.8	[M+H]	500.3	y4	15.5	7.1
P11137	*MAP2*	LINQPLPDLK	[M+2H]	575.8	[M+H]	682.4	y6	18.9	11.4
			[M+2H]	575.8	[M+H]	472.3	y4	18.9	11.4
			[M+2H]	575.8	[M+H]	260.2	y2	18.9	11.4
		LINQPLPDL**K**	**[M+2H]**	**579.9**	**[M+H]**	**690.4**	**y6**	**18.9**	**11.4**

			[M+2H]	579.9	[M+H]	480.3	y4	18.9	11.4
			[M+2H]	579.9	[M+H]	268.2	y2	18.9	11.4
		LASVSADAEVAR	[M+2H]	594.8	[M+H]	818.4	y8	19.4	5.9
			[M+2H]	594.8	[M+H]	731.4	y7	19.4	5.9
			[M+2H]	594.8	[M+H]	660.3	y6	19.4	5.9
		LASVSADAEVA**R**	[M+2H]	599.8	[M+H]	828.4	y8	19.4	5.9
			[M+2H]	599.8	[M+H]	741.4	y7	19.4	5.9
			[M+2H]	599.8	[M+H]	670.3	y6	19.4	5.9
		VDHGAEIITQSPGR	[M+3H]	493.9	[M+H]	645.3	y6	13	4.6
			[M+3H]	493.9	[M+H]	544.3	y5	13	4.6
			[M+3H]	493.9	[M+H]	416.2	y4	13	4.6
		VDHGAEIITQSPG**R**	[M+3H]	497.3	[M+H]	655.3	y6	13	4.6
			[M+3H]	497.3	[M+H]	554.3	y5	13	4.6
			[M+3H]	497.3	[M+H]	426.2	y4	13	4.6
P17600	*SYN1*	SLKPDFVLIR	[M+3H]	396.6	[M+H]	500.4	y4	9.5	12.5
			[M+3H]	396.6	[M+H]	401.3	y3	9.5	12.5
			[M+3H]	396.6	[M+H]	688.4	b6	9.5	12.5
		SLKPDFVLI**R**	[M+3H]	399.9	[M+H]	510.4	y4	9.5	12.5
			**[M+3H]**	**399.9**	**[M+H]**	**411.3**	**y3**	**9.5**	**12.5**
			[M+3H]	399.9	[M+H]	688.4	b6	9.5	12.5
		GSHGQTPSPGALPLGR	[M+3H]	511.3	[M+H]	780.5	y8	13.6	6.4
			[M+3H]	511.3	[M+H]	442.3	y4	13.6	6.4
			[M+3H]	511.3	[M+2H]	390.7	y8	13.6	6.4
		GSHGQTPSPGALPLG**R**	[M+3H]	514.6	[M+H]	790.5	y8	13.6	6.4
			[M+3H]	514.6	[M+H]	452.3	y4	13.6	6.4
			[M+3H]	514.6	[M+2H]	395.7	y8	13.6	6.4
P04271	*S100B*	AMVALIDVFHQYSGR	[M+3H]	569.6	[M+2H]	752.9	y13	15.7	18.9
			[M+3H]	569.6	[M+2H]	703.4	y12	15.7	18.9
			[M+3H]	569.6	[M+2H]	667.8	y11	15.7	18.9
		AMVALIDVFHQYSG**R**	[M+3H]	573.0	[M+2H]	757.9	y13	15.7	18.9
			**[M+3H]**	**573.0**	**[M+2H]**	**708.4**	**y12**	**15.7**	**18.9**

			[M+3H]	573.0	[M+2H]	672.9	y11	15.7	18.9
		EQEVVDK	[M+2H]	423.7	[M+H]	589.3	y5	14.1	1.2
			[M+2H]	423.7	[M+H]	460.3	y4	14.1	1.2
			[M+2H]	423.7	[M+H]	361.2	y3	14.1	1.2
		EQEVVD**K**	[M+2H]	427.7	[M+H]	597.3	y5	14.1	1.2
			[M+2H]	427.7	[M+H]	468.3	y4	14.1	1.2
			[M+2H]	427.7	[M+H]	369.2	y3	14.1	1.2
P14136	*GFAP*	HLQEYQDLLNVK	[M+3H]	500.6	[M+H]	701.4	y6	13.2	11.3
			[M+3H]	500.6	[M+H]	473.3	y4	13.2	11.3
			[M+3H]	500.6	[M+H]	508.3	b4	13.2	11.3
		HLQEYQDLLNV**K**	[M+3H]	503.3	[M+H]	709.4	y6	13.2	11.3
			[M+3H]	503.3	[M+H]	481.3	y4	13.2	11.3
			[M+3H]	503.3	[M+H]	508.3	b4	13.2	11.3
		QLQSLTC[+57]DLESLR	[M+2H]	781.9	[M+H]	1106.6	y9	25.2	16.3
			[M+2H]	781.9	[M+H]	993.5	y8	25.2	16.3
			[M+2H]	781.9	[M+H]	892.4	y7	25.2	16.3
		QLQSLTC[+57]DLESL**R**	[M+2H]	786.9	[M+H]	1116.6	y9	25.2	16.3
			[M+2H]	786.9	[M+H]	1003.5	y8	25.2	16.3
			[M+2H]	786.9	[M+H]	902.4	y7	25.2	16.3
		DNLAQDLATVR	[M+2H]	608.3	[M+H]	873.5	y8	19.9	13.2
			[M+2H]	608.3	[M+H]	802.4	y7	19.9	13.2
			[M+2H]	608.3	[M+H]	230.1	b2	19.9	13.2
		DNLAQDLATV**R**	[M+2H]	613.3	[M+H]	883.5	y8	19.9	13.2
			[M+2H]	613.3	[M+H]	812.5	y7	19.9	13.2
			**[M+2H]**	**613.3**	**[M+H]**	**230.1**	**b2**	**19.9**	**13.2**
P16070	*CD44*	YGFIEGHVVIPR	[M+3H]	462.9	[M+H]	583.4	y5	11.9	12.3
			[M+3H]	462.9	[M+2H]	612.3	y11	11.9	12.3
			[M+3H]	462.9	[M+2H]	510.3	y9	11.9	12.3
		YGFIEGHVVIP**R**	[M+3H]	466.3	[M+H]	593.4	y5	11.9	12.3
			[M+3H]	466.3	[M+2H]	617.4	y11	11.9	12.3
			[M+3H]	466.3	[M+2H]	515.3	y9	11.9	12.3

		ALSIGFETC[+57]R	[M+2H]	577.3	[M+H]	969.4	y8	18.9	11.1
			[M+2H]	577.3	[M+H]	769.3	y6	18.9	11.1
			[M+2H]	577.3	[M+H]	565.2	y4	18.9	11.1
		ALSIGFETC[+57]**R**	[M+2H]	582.3	[M+H]	979.5	y8	18.9	11.1
			**[M+2H]**	**582.3**	**[M+H]**	**779.3**	**y6**	**18.9**	**11.1**
			[M+2H]	582.3	[M+H]	575.2	y4	18.9	11.1
		FAGVFHVEK	[M+3H]	345.2	[M+H]	375.2	y3	7.6	6.9
			[M+3H]	345.2	[M+2H]	443.7	y8	7.6	6.9
			[M+3H]	345.2	[M+2H]	330.2	y5	7.6	6.9
		FAGVFHVE**K**	[M+3H]	347.9	[M+H]	383.2	y3	7.6	6.9
			[M+3H]	347.9	[M+2H]	447.7	y8	7.6	6.9
			[M+3H]	347.9	[M+2H]	334.2	y5	7.6	6.9
		TEAADLC[+57]K	[M+2H]	454.2	[M+H]	677.3	y6	15.1	1.6
			[M+2H]	454.2	[M+H]	535.3	y4	15.1	1.6
			[M+2H]	454.2	[M+H]	420.2	y3	15.1	1.6
		TEAADLC[+57]**K**	[M+2H]	458.2	[M+H]	685.3	y6	15.1	1.6
			[M+2H]	458.2	[M+H]	543.3	y4	15.1	1.6
			[M+2H]	458.2	[M+H]	428.2	y3	15.1	1.6
P02766	*TTR*	AADDTWEPFASGK	[M+2H]	697.8	[M+H]	735.4	y7	22.6	12.2
			[M+2H]	697.8	[M+H]	606.3	y6	22.6	12.2
			[M+2H]	697.8	[M+H]	362.2	y4	22.6	12.2
		AADDTWEPFASG**K**	[M+2H]	701.8	[M+H]	743.4	y7	22.6	12.2
			**[M+2H]**	**701.8**	**[M+H]**	**614.3**	**y6**	**22.6**	**12.2**
			[M+2H]	701.8	[M+H]	370.2	y4	22.6	12.2
		GSPAINVAVHVF**R**	[M+3H]	456.3	[M+H]	728.4	y6	11.6	12.0
			[M+3H]	456.3	[M+H]	558.3	y4	11.6	12.0
			[M+3H]	456.3	[M+2H]	611.9	y11	11.6	12.0
		GSPAINVAVHVF**R**	[M+3H]	459.6	[M+H]	738.4	y6	11.6	12.0
			[M+3H]	459.6	[M+H]	568.3	y4	11.6	12.0
			[M+3H]	459.6	[M+2H]	616.9	y11	11.6	12.0
P60709	*ACTB*	AVFPSIVGR	[M+2H]	473.3	[M+H]	775.4	y7	15.7	12.0

			[M+2H]	473.3	[M+H]	628.4	y6	15.7	12.0
			[M+2H]	473.3	[M+2H]	314.7	y6	15.7	12.0
		AVFPSIVG**R**	**[M+2H]**	**478.3**	**[M+H]**	**785.5**	**y7**	**15.7**	**12.0**
			[M+2H]	478.3	[M+H]	638.4	y6	15.7	12.0
			[M+2H]	478.3	[M+2H]	319.7	y6	15.7	12.0
		EITALAPSTMK	[M+2H]	581.3	[M+H]	747.4	y7	19	8.9
			[M+2H]	581.3	[M+H]	634.3	y6	19	8.9
			[M+2H]	581.3	[M+H]	563.3	y5	19	8.9
		EITALAPSTM**K**	[M+2H]	585.3	[M+H]	755.4	y7	19	8.9
			[M+2H]	585.3	[M+H]	642.3	y6	19	8.9
			[M+2H]	585.3	[M+H]	571.3	y5	19	8.9
P04406	*GAPDH*	LVINGNPITIFQER	[M+2H]	807.5	[M+H]	1003.6	y8	26	19.1
			[M+2H]	807.5	[M+H]	793.4	y6	26	19.1
			[M+2H]	807.5	[M+H]	579.3	y4	26	19.1
		LVINGNPITIFQE**R**	[M+2H]	812.5	[M+H]	1013.6	y8	26	19.1
			[M+2H]	812.5	[M+H]	803.4	y6	26	19.1
			[M+2H]	812.5	[M+H]	589.3	y4	26	19.1
		LISWYDNEFGYSNR	[M+2H]	882.4	[M+H]	1264.5	y10	28.4	16.7
			[M+2H]	882.4	[M+H]	1101.5	y9	28.4	16.7
			[M+2H]	882.4	[M+H]	743.3	y6	28.4	16.7
		LISWYDNEFGYSN**R**	[M+2H]	887.4	[M+H]	1274.5	y10	28.4	16.7
			**[M+2H]**	**887.4**	**[M+H]**	**1111.5**	**y9**	**28.4**	**16.7**
			[M+2H]	887.4	[M+H]	753.4	y6	28.4	16.7
P07737	*PFN1*	STGGAPTFNVTVTK	[M+2H]	690.4	[M+H]	1006.6	y9	22.4	9.3
			[M+2H]	690.4	[M+2H]	503.8	y9	22.4	9.3
			[M+2H]	690.4	[M+H]	374.2	b5	22.4	9.3
		STGGAPTFNVTVT**K**	[M+2H]	694.4	[M+H]	1014.6	y9	22.4	9.3
			[M+2H]	694.4	[M+2H]	507.8	y9	22.4	9.3
			[M+2H]	694.4	[M+H]	374.2	b5	22.4	9.3
		DSPSVWAAVPGK	[M+2H]	607.3	[M+H]	728.4	y7	19.8	11.8
			[M+2H]	607.3	[M+H]	301.2	y3	19.8	11.8

			[M+2H]	607.3	[M+2H]	506.3	y10	19.8	11.8
		DSPSVWAAVPG**K**	[M+2H]	611.3	[M+H]	736.4	y7	19.8	11.8
			**[M+2H]**	**611.3**	**[M+H]**	**309.2**	**y3**	**19.8**	**11.8**
			[M+2H]	611.3	[M+2H]	510.3	y10	19.8	11.8
P40227	*CCT6A*	ALQFLEEVK	[M+2H]	538.8	[M+H]	892.5	y7	17.7	13.9
			[M+2H]	538.8	[M+H]	764.4	y6	17.7	13.9
			[M+2H]	538.8	[M+H]	617.4	y5	17.7	13.9
			[M+2H]	538.8	[M+H]	504.3	y4	17.7	13.9
		ALQFLEEV**K**	[M+2H]	542.8	[M+H]	900.5	y7	17.7	13.9
			**[M+2H]**	**542.8**	**[M+H]**	**772.4**	**y6**	**17.7**	**13.9**
			[M+2H]	542.8	[M+H]	625.4	y5	17.7	13.9
			[M+2H]	542.8	[M+H]	512.3	y4	17.7	13.9
Q99729	*HNRNPAB*	GFVFITFK	[M+2H]	479.8	[M+H]	754.4	y6	15.9	18.1
			[M+2H]	479.8	[M+H]	655.4	y5	15.9	18.1
			[M+2H]	479.8	[M+H]	205.1	b2	15.9	18.1
		GFVFITF**K**	[M+2H]	483.8	[M+H]	762.5	y6	15.9	18.1
			**[M+2H]**	**483.8**	**[M+H]**	**663.4**	**y5**	**15.9**	**18.1**
			[M+2H]	483.8	[M+H]	205.1	b2	15.9	18.1
		FHTVSGSK	[M+3H]	288.2	[M+H]	378.2	y4	5.6	0.8
			[M+3H]	288.2	[M+H]	291.2	y3	5.6	0.8
			[M+3H]	288.2	[M+H]	386.2	b3	5.6	0.8
		FHTVSGS**K**	[M+3H]	290.8	[M+H]	386.2	y4	5.6	0.8
			[M+3H]	290.8	[M+H]	299.2	y3	5.6	0.8
			[M+3H]	290.8	[M+H]	386.2	b3	5.6	0.8
P51665	*PSMD7*	VVGVLLGSWQK	[M+2H]	593.4	[M+H]	987.6	y9	19.4	14.6
			[M+2H]	593.4	[M+H]	831.5	y7	19.4	14.6
			[M+2H]	593.4	[M+H]	605.3	y5	19.4	14.6
		VVGVLLGSWQ**K**	**[M+2H]**	**597.4**	**[M+H]**	**995.6**	**y9**	**19.4**	**14.6**
			[M+2H]	597.4	[M+H]	839.5	y7	19.4	14.6
			[M+2H]	597.4	[M+H]	613.3	y5	19.4	14.6
		IVGWYHTGPK	[M+3H]	386.5	[M+H]	702.4	y6	9.1	5.6

	[M+3H]	386.5	[M+2H]	473.2	y8	9.1	5.6
	[M+3H]	386.5	[M+H]	213.2	b2	9.1	5.6
IVGWYHTGP**K**	[M+3H]	389.2	[M+H]	710.4	y6	9.1	5.6
	[M+3H]	389.2	[M+2H]	477.2	y8	9.1	5.6
	[M+3H]	389.2	[M+H]	213.2	b2	9.1	5.6

Table S5. Calibration curve parameters **(a)**, linearity range, the limit of detection/quantification (LOD/LOQ), coefficient of variation (CV%) for ganglioside assays **(b)**

(a)

	Calibration parameters									
**Calibration points**	1	2	3	4	5	6	7	8	9	10
**GM3 Concentration (nM)**	2.54	7.62	22.86	68.59	205.76	617.28	1851.85	5555.56	16666.67	50000
**GM1 Concentration (nM)**	0.03	0.091	0.274	0.823	2.47	7.41	22.22	66.67	200	600
**Number of technical replicates**	4	4	4	4	3	3	3	3	3	3

(b)

Parameters	GM1	GM3	GT1b	GD1b	GD2	GD3	GD1a	GM2
**min conc. in sample [nM]**	14.04	110.52	51.62	88.30	170.08	4787.27	73.22	20.26
**max conc. in sample [nM]**	876.20	1016.58	4501.39	3826.64	5521.14	46068.99	4346.07	1180.07
**LOD [nM]**	3.19	8.19	5.43	3.62	1.71	41.33	2.81	1.40
**LOQ [nM]**	10.62	27.29	18.11	12.07	5.71	137.76	9.35	4.67
**Calibration range [points]**	1 - 7	1 - 9	n.a.*	n.a.	n.a.	n.a.	n.a.	n.a.
**R^2**	0.9999	0.9974	n.a.	n.a.	n.a.	n.a.	n.a.	n.a.
**CV [%]**	3.5	3.4	2.1	2.9	4.9	12.1	2.0	4.1

* n.a. = not available. The calibration curves were prepared for GM1 and GM3 isotopically-labeled internal standards. The LOD and LOQ values for other GSs were determined via the response factor.

Table S6. Ganglioside internal standards: signal reproducibility, recovery, and matrix effects

**GS**	**signal stability** in solvent **CV [%]**	**signal stability** in matrix **CV [%]**	**Recovery** in matrix **[%]**	**matrix effect** in matrix* **[%]**
13C_GM3	3.8	3.6	82.3	108.9
13C_GM1	2.9	6	105.1	110.7

*matrix effect was determined by the ratio of recovery of internal standards signal in a neat solvent to the recovery of internal standard in the sample matrix.

Table S7. Calibration curve parameters **(a)**, linearity range, the limit of detection/quantification (LOD/LOQ), coefficient of variation (CV%) for protein assays **(b)**

(a)

	Calibration parameters									
**Calibration points**	1	2	3	4	5	6	7	8	9	10
**Concentration (nM)**	1.02	5.08	10.16	20.31	40.63	81.25	162.50	325.00	650.00	1300.00
**Number of technical replicates**	6	6	6	6	4	4	4	4	4	4

(b)

**Parameters**	**Protein**	**SOX2**	**FABP7**	**DCX**	**TUBB3**	**MAP2**	**NEFL**	**NEFM**	**S100B**	**GFAP**	**CD44**	**TTR**	**ACTB**	**GAPDH**	**SYN1**
	**Quantifier ST peptides**	LLSETEK	ALGVGFATR	YIYTIDGSR	ISVYYNEASSHK	LINQPLPDLK	FTVLTESAAK	SIELESVR	AMVALIDVFHQYSGR	DNLAQDLATVR	ALSIGFETCR	AADDTWEPFASGK	AVFPSIVGR	LISWYDNEFGYSNR	SLKPDFVLIR
**min conc. in sample [nM]**		1.1	5.8	1.4	5.1	3.0	0.4	1.1	1.7	0.2	0.3	1.3	62.1	64.7	0.1
**max conc. in sample [nM]**		7.8	121.7	13.1	49.9	25.1	4.8	10.7	108.8	9.9	17.8	37.2	796.8	452.2	0.9
**Concentration in QC sample [nM]**		1.96	23.07	2.99	16.19	5.90	0.86	3.43	9.49	n.d.	1.32	15.67	199.14	192.34	n.d.
**LOD**															
**Concentration [nM]**		0.33	0.95	1.90	2.84	0.27	0.40	0.69	0.84	0.19	0.28	1.40	0.44	1.40	0.56
**Concentration [pg/ug of total protein]**		5.6	7.1	38.6	71.7	27.1	12.2	35.3	4.5	4.6	11.4	11.1	9.1	25.2	20.6
**Theoretical total protein amount needed for molar LOD**		5.0	1.2	**19.1**	5.3	1.4	13.8	6.0	2.7	n. d.	6.4	2.7	**0.1**	0.2	n.d.
**LOQ**															
**Concentration [nM]**		1.09	3.17	3.18	9.48	0.90	1.32	2.30	2.81	0.62	0.93	4.67	1.46	4.65	1.85
**Concentration [pg/ug of total protein]**		18.7	23.6	64.6	239.1	90.3	40.6	117.7	15.1	15.4	38.1	37.1	30.5	83.9	68.7
**Theoretical total protein amount needed for molar LOQ**		16.7	4.1	32.0	17.6	4.6	**45.9**	20.1	8.9	n. d.	21.3	9.0	**0.2**	0.7	n.d.
**Calibration range [points]**		1 - 6	1 - 8	1 - 8	2 - 8	1 - 8	1 - 8	1 - 8	1 - 8	1 - 8	1 - 8	1 - 8	1 - 10	1 - 10	2 - 8
**R^2**		0.9985	0.9993	0.9993	0.9994	0.9988	0.9968	0.9998	0.9983	0.9989	0.9998	0.9988	0.9982	0.9990	0.9988
**CV [%]**		12	9	13	12	11	17	11	10	6	11	12	9	7	21

Table S8. The variability in quantitation of protein and lipid markers before **(a)** and after **(b)** removing outlier cerebral organoids **(c)** data shows improved %CV by up to 26 %.

(a)

Biological variability (%CV) of proteins and GSs **before** sorting, **n = 4**

%CV in each timepoint (n = 4)	TTR	SOX2	FABP7	DCX	TUBB3	MAP2	NEFL	NEFM	S100B	GFAP	CD44	ACTB	GAPDH	GT1b	GD1b	GD2	GD3	GD1a	GM3	GM1	GM2
**48 day**	50.9	38.8	38.3	11.5	18.6	28.0	40.5	31.5	50.5	0.0	173.2	52.8	31.6	22.4	32.0	44.0	36.4	33.9	45.2	33.2	49.8
**76 day**	59.5	38.9	21.1	20.8	38.0	39.4	54.0	40.7	102.1	173.2	118.3	97.3	56.7	37.7	37.9	26.2	33.4	36.7	50.2	45.2	14.6
**95 day**	79.8	43.0	32.2	27.9	24.6	44.5	30.8	26.8	24.9	59.1	34.3	87.1	83.0	39.7	40.9	74.0	44.2	34.1	55.2	42.7	83.7
**110 day**	68.4	34.5	28.1	24.4	39.9	32.6	29.6	19.4	38.4	112.9	44.4	43.3	69.8	28.3	33.6	24.9	7.5	25.6	11.7	33.3	24.9
**135 day**	116.7	60.0	21.7	53.1	23.5	65.0	32.9	44.1	10.1	49.2	48.2	51.5	60.3	89.2	78.6	89.5	99.9	78.3	107.2	64.7	73.4
**160 day**	58.3	30.5	34.2	22.7	24.0	26.7	35.5	23.5	35.0	53.6	37.8	23.7	44.1	39.2	33.6	28.0	34.0	44.4	25.3	37.4	27.2

(b)

Biological variability (%CV) of proteins and GSs **after** sorting, **n = 3**

%CV in each timepoint (n = 3)	TTR	SOX2	FABP7	DCX	TUBB3	MAP2	NEFL	NEFM	S100B	GFAP	CD44	ACTB	GAPDH	GT1b	GD1b	GD2	GD3	GD1a	GM3	GM1	GM2
**48 day**	25.5	16.8	5.3	13.0	20.2	30.3	26.5	31.1	25.4	0.0	141.4	19.4	22.4	22.9	28.6	19.5	11.6	23.4	13.6	29.2	28.7
**76 day**	28.8	18.2	14.8	6.3	24.6	12.4	43.5	26.5	85.1	141.4	95.6	31.6	36.4	41.2	41.1	23.4	14.0	33.1	29.6	42.0	16.6
**95 day**	74.2	51.2	37.6	28.5	23.9	31.7	22.1	25.5	9.0	72.1	21.4	91.0	102.6	44.6	41.7	24.7	41.4	40.1	66.7	43.1	32.3
**110 day**	69.4	37.0	32.8	28.5	34.0	37.7	17.9	21.4	37.2	84.1	34.8	38.5	55.4	33.1	37.2	23.6	6.3	29.2	7.2	32.4	24.7
**135 day**	39.6	6.2	23.2	26.9	25.3	30.3	31.3	44.4	10.8	27.2	15.3	19.2	45.1	47.9	71.6	24.3	13.8	58.9	14.3	61.7	33.9
**160 day**	60.1	8.6	22.3	18.3	26.8	7.9	24.7	9.0	15.9	45.3	34.7	22.2	47.4	25.3	20.1	15.4	23.0	29.2	13.6	24.2	10.2

(c)

Effect of the CO elimination process on the biological variability of proteins and GSs

Average of %CV within the whole timeline	TTR	SOX2	FABP7	DCX	TUBB3	MAP2	NEFL	NEFM	S100B	GFAP	CD44	ACTB	GAPDH	GT1b	GD1b	GD2	GD3	GD1a	GM3	GM1	GM2
**Before sorting (n = 4)**	72.3	40.9	29.3	26.7	28.1	39.4	37.2	31.0	43.5	74.7	76.0	59.3	57.6	42.8	42.8	47.8	42.6	42.2	49.1	42.7	45.6
**After sorting (n = 3)**	49.6	23.0	22.7	20.3	25.8	25.0	27.7	26.3	30.6	61.7	57.2	37.0	51.5	35.8	40.0	21.8	18.4	35.7	24.2	38.8	24.4
**Difference**	22.7	17.9	6.6	6.5	2.3	14.3	9.5	4.7	12.9	13.0	18.8	22.3	6.0	6.9	2.7	26.0	24.2	6.5	25.0	4.0	21.2

Table S9. The algorithm to assess heterogeneity in cerebral organoids (n=4 at each time point)

SAMPLES [Day of CO]	NEURONS to GLIAL CELLS ratio (NGR)	MED	35 % of MED	min	max	Sum of all prot. marker levels (PML)	MED	35 % of MED	min	max	Sum of all GSs/lipid marker levels (LML)	MED	35 % of MED	min	max	SCORE
**D48_1**	8.3	10.4	3.7	6.8	14.1	280.7	357.4	125.1	232.3	482.5	21110.5	23382.9	8184.0	15198.9	31567.0	**0**
**D48_2**	12.6					418.8					27477.1					**0**
**D48_3**	12.5					425.9					25655.3					**0**
**D48_4**	6.6					296.0					8443.0					**3**
**D76_1**	7.7	6.4	2.2	4.1	8.6	372.2	419.5	146.8	272.7	566.4	14461.5	12451.5	4358.0	8093.5	16809.5	**0**
**D76_2**	3.9					163.5					6098.2					**4**
**D76_3**	7.5					517.9					14607.4					**0**
**D76_4**	5.2					466.8					10441.5					**0**
**D95_1**	2.9	3.3	1.2	2.2	4.5	724.8	963.4	337.2	626.2	1300.6	30754.2	38888.8	13611.1	25277.7	52499.8	**0**
**D95_2**	3.5					719.5					17512.3					**1**
**D95_3**	3.1					1329.2					47023.3					**1**
**D95_4**	5.1					1202.0					62826.8					**3**
**D110_1**	2.1	2.6	0.9	1.7	3.5	987.8	1164.0	407.4	756.6	1571.3	43090.4	43177.9	15112.3	28065.7	58290.2	**0**
**D110_2**	2.1					836.2					31771.4					**0**
**D110_3**	3.8					1340.1					47587.7					**2**
**D110_4**	3.0					1398.8					43265.4					**0**
**D135_1**	3.7	3.8	1.3	2.5	5.2	1000.9	834.4	292.0	542.3	1126.4	28916.7	24906.2	8717.2	16189.0	33623.4	**0**
**D135_2**	2.6					598.1					18847.2					**0**
**D135_3**	9.9					1629.3					137240.5					**4**
**D135_4**	3.9					667.8					20895.7					**0**
**D160_1**	2.9	2.4	0.8	1.6	3.3	639.0	1143.9	400.3	743.5	1544.2	24728.8	44201.7	15470.6	28731.1	59672.2	**2**
**D160_2**	1.6					1203.7					61750.8					**1**
**D160_3**	1.9					1151.5					43301.7					**0**
**D160_4**	3.3					1136.2					45101.6					**0**

Scoring parameters were: i) neurons to glia ratio (NGR) = the sum of DCX, TUBB3, MAP2, and NEFM to the sum of FABP7 and S100B; ii) sum of all protein markers and housekeeping proteins ACTB and GAPDH levels except for SYN1 (PML), and the sum of eight GSs levels (LML). The upper and lower cut-off range was determined using median value +/- 35 %. COs with NGR, PML, or LML values below or above the cut-off range were marked (in red). NGR parameter outside the median +/- 35 % received the score of two, PML or LML outside the range received the score one (Table S10). The highest scoring COs were excessively variable and eliminated from further interpretation.

Table S10. A score to assess potential sources of heterogeneity in cerebral organoids

Sorting Score	Interpretation
0	Similar COs
1	Varying differentiation efficiency
2	Varying differentiation efficiency or cellular composition
3,4	Varying differentiation efficiency and cellular composition

Table S11. The list of correlation coefficients and p-values for protein and lipid markers across **(a)** early (D48, D75, D96) and **(b)** later (D110, D135, D160) time points. Significant differences have been highlighted.

(a)

Correlation coefficients

	SOX2	GM3	GD3	TTR	CD44	MAP2	S100B	FABP7	TUBB3	NEFL	NEFM	GD1a	GT1b	GD1b	GM1	DCX	GM2	GD2
**SOX2**	1	0.5285	0.52566	0.20567	-0.48499	-0.24672	-0.43734	-0.37909	-0.45311	-0.24856	-0.1955	-0.30084	-0.36213	-0.30901	-0.3343	-0.39156	-0.2883	-0.31584
**GM3**	0.5285	1	0.90746	0.9301	0.17044	0.45509	0.17192	0.49236	0.33979	0.47298	0.54758	0.28139	0.18279	0.20595	0.32889	0.35049	0.47584	0.45251
**GD3**	0.52566	0.90746	1	0.84902	0.22281	0.52793	0.28443	0.572	0.47602	0.51121	0.61587	0.49751	0.41545	0.45127	0.52781	0.52985	0.62394	0.60893
**TTR**	0.20567	0.9301	0.84902	1	0.44196	0.69086	0.38619	0.74461	0.59575	0.68308	0.75674	0.42375	0.33344	0.34386	0.50212	0.56923	0.67378	0.67469
**CD44**	-0.48499	0.17044	0.22281	0.44196	1	0.7298	0.81843	0.78272	0.68357	0.73924	0.73717	0.52487	0.48086	0.44265	0.60426	0.67847	0.69998	0.68992
**MAP2**	-0.24672	0.45509	0.52793	0.69086	0.7298	1	0.5498	0.87042	0.83822	0.806	0.86314	0.52017	0.49367	0.54425	0.64447	0.70877	0.74715	0.75849
**S100B**	-0.43734	0.17192	0.28443	0.38619	0.81843	0.5498	1	0.78037	0.73226	0.78578	0.77429	0.80141	0.7627	0.70661	0.80122	0.83835	0.84992	0.82406
**FABP7**	-0.37909	0.49236	0.572	0.74461	0.78272	0.87042	0.78037	1	0.95688	0.87467	0.92024	0.79709	0.76124	0.75451	0.87253	0.93172	0.95359	0.95504
**TUBB3**	-0.45311	0.33979	0.47602	0.59575	0.68357	0.83822	0.73226	0.95688	1	0.85575	0.88224	0.83603	0.83677	0.84719	0.90227	0.93118	0.92804	0.92852
**NEFL**	-0.24856	0.47298	0.51121	0.68308	0.73924	0.806	0.78578	0.87467	0.85575	1	0.9813	0.64216	0.60744	0.60674	0.70512	0.76208	0.83104	0.83099
**NEFM**	-0.1955	0.54758	0.61587	0.75674	0.73717	0.86314	0.77429	0.92024	0.88224	0.9813	1	0.6797	0.63546	0.64109	0.74773	0.81637	0.88155	0.89254
**GD1a**	-0.30084	0.28139	0.49751	0.42375	0.52487	0.52017	0.80141	0.79709	0.83603	0.64216	0.6797	1	0.99088	0.97437	0.98618	0.95114	0.92749	0.88435
**GT1b**	-0.36213	0.18279	0.41545	0.33344	0.48086	0.49367	0.7627	0.76124	0.83677	0.60744	0.63546	0.99088	1	0.98926	0.97534	0.92986	0.89058	0.84869
**GD1b**	-0.30901	0.20595	0.45127	0.34386	0.44265	0.54425	0.70661	0.75451	0.84719	0.60674	0.64109	0.97437	0.98926	1	0.96934	0.91244	0.87639	0.83084
**GM1**	-0.3343	0.32889	0.52781	0.50212	0.60426	0.64447	0.80122	0.87253	0.90227	0.70512	0.74773	0.98618	0.97534	0.96934	1	0.97652	0.95908	0.919
**DCX**	-0.39156	0.35049	0.52985	0.56923	0.67847	0.70877	0.83835	0.93172	0.93118	0.76208	0.81637	0.95114	0.92986	0.91244	0.97652	1	0.98592	0.97651
**GM2**	-0.2883	0.47584	0.62394	0.67378	0.69998	0.74715	0.84992	0.95359	0.92804	0.83104	0.88155	0.92749	0.89058	0.87639	0.95908	0.98592	1	0.98505
**GD2**	-0.31584	0.45251	0.60893	0.67469	0.68992	0.75849	0.82406	0.95504	0.92852	0.83099	0.89254	0.88435	0.84869	0.83084	0.919	0.97651	0.98505	1

P-value

	SOX2	GM3	GD3	TTR	CD44	MAP2	S100B	FABP7	TUBB3	NEFL	NEFM	GD1a	GT1b	GD1b	GM1	DCX	GM2	GD2
**SOX2**	NA	0.14353	0.1461	0.59549	0.18574	0.5222	0.23913	0.31433	0.22063	0.519	0.6142	0.4315	0.33822	0.41845	0.37925	0.29733	0.45186	0.40768
**GM3**	0.14353	NA	0.000724	0.000278	0.66108	0.21837	0.65828	0.17815	0.37098	0.19849	0.12699	0.46324	0.63783	0.595	0.38748	0.3551	0.19541	0.22133
**GD3**	0.1461	0.000724	NA	0.003786	0.56446	0.14405	0.45822	0.10755	0.19521	0.15956	0.077423	0.17295	0.26612	0.22275	0.14415	0.14232	0.072529	0.081787
**TTR**	0.59549	0.000278	0.003786	NA	0.23363	0.039339	0.30459	0.021364	0.090491	0.042551	0.018254	0.25571	0.38055	0.3649	0.16837	0.10966	0.046604	0.046199
**CD44**	0.18574	0.66108	0.56446	0.23363	NA	0.025614	0.006997	0.012636	0.042343	0.022847	0.023435	0.14681	0.19007	0.23281	0.084809	0.044532	0.035778	0.039715
**MAP2**	0.5222	0.21837	0.14405	0.039339	0.025614	NA	0.12514	0.002267	0.004769	0.008709	0.002724	0.15113	0.17682	0.12979	0.060972	0.032549	0.020686	0.017832
**S100B**	0.23913	0.65828	0.45822	0.30459	0.006997	0.12514	NA	0.013088	0.024874	0.012063	0.01431	0.009406	0.016842	0.033326	0.009436	0.004756	0.003712	0.006304
**FABP7**	0.31433	0.17815	0.10755	0.021364	0.012636	0.002267	0.013088	NA	5.26E-05	0.002026	0.000436	0.010097	0.017181	0.018802	0.002145	0.000256	6.77E-05	6.07E-05
**TUBB3**	0.22063	0.37098	0.19521	0.090491	0.042343	0.004769	0.024874	5.26E-05	NA	0.00325	0.001642	0.004987	0.004912	0.003942	0.000872	0.000263	0.000307	0.0003
**NEFL**	0.519	0.19849	0.15956	0.042551	0.022847	0.008709	0.012063	0.002026	0.00325	NA	2.89E-06	0.062212	0.082742	0.083192	0.033866	0.016986	0.005511	0.005516
**NEFM**	0.6142	0.12699	0.077423	0.018254	0.023435	0.002724	0.01431	0.000436	0.001642	2.89E-06	NA	0.043996	0.065888	0.062787	0.020533	0.007263	0.001674	0.001204
**GD1a**	0.4315	0.46324	0.17295	0.25571	0.14681	0.15113	0.009406	0.010097	0.004987	0.062212	0.043996	NA	2.37E-07	8.66E-06	1.01E-06	8.09E-05	0.000315	0.001544
**GT1b**	0.33822	0.63783	0.26612	0.38055	0.19007	0.17682	0.016842	0.017181	0.004912	0.082742	0.065888	2.37E-07	NA	4.18E-07	7.57E-06	0.000281	0.00128	0.003814
**GD1b**	0.41845	0.595	0.22275	0.3649	0.23281	0.12979	0.033326	0.018802	0.003942	0.083192	0.062787	8.66E-06	4.18E-07	NA	1.61E-05	0.0006	0.001934	0.005533
**GM1**	0.37925	0.38748	0.14415	0.16837	0.084809	0.060972	0.009436	0.002145	0.000872	0.033866	0.020533	1.01E-06	7.57E-06	1.61E-05	NA	6.38E-06	4.39E-05	0.00046
**DCX**	0.29733	0.3551	0.14232	0.10966	0.044532	0.032549	0.004756	0.000256	0.000263	0.016986	0.007263	8.09E-05	0.000281	0.0006	6.38E-06	NA	1.08E-06	6.39E-06
**GM2**	0.45186	0.19541	0.072529	0.046604	0.035778	0.020686	0.003712	6.77E-05	0.000307	0.005511	0.001674	0.000315	0.00128	0.001934	4.39E-05	1.08E-06	NA	1.32E-06
**GD2**	0.40768	0.22133	0.081787	0.046199	0.039715	0.017832	0.006304	6.07E-05	0.0003	0.005516	0.001204	0.001544	0.003814	0.005533	0.00046	6.39E-06	1.32E-06	NA

(b)

Correlation coefficients

	DCX	TUBB3	GM3	TTR	FABP7	NEFL	NEFM	GD1b	GM1	GT1b	GD1a	CD44	GFAP	S100B	GM2	GD3	GD2	MAP2	SOX2
**DCX**	1	0.86151	0.47148	0.21301	0.64878	0.81996	0.72283	0.8044	0.71703	0.57741	0.60955	-0.52803	-0.64032	-0.22592	-0.07377	-0.17803	-0.25086	0.40249	0.16624
**TUBB3**	0.86151	1	0.21779	-0.25287	0.61287	0.70025	0.65319	0.72928	0.64225	0.4787	0.50452	-0.57728	-0.58057	-0.28877	0.10087	-0.28766	-0.10313	0.60257	0.17048
**GM3**	0.47148	0.21779	1	0.57674	0.55333	0.74469	0.69902	0.61187	0.61926	0.59412	0.62144	0.25347	-0.02065	0.69972	0.50988	0.64194	0.27587	0.36529	0.5813
**TTR**	0.21301	-0.25287	0.57674	1	0.020527	0.34242	0.26086	0.151	0.18898	0.27063	0.28731	0.099107	-0.17413	0.31338	-0.08817	0.25645	-0.12688	-0.29851	-0.09372
**FABP7**	0.64878	0.61287	0.55333	0.020527	1	0.71036	0.63977	0.72882	0.62074	0.42354	0.47833	-0.1008	0.025711	0.19377	0.31314	0.29109	0.027783	0.61006	0.5663
**NEFL**	0.81996	0.70025	0.74469	0.34242	0.71036	1	0.93698	0.88893	0.88565	0.79758	0.84535	-0.40519	-0.53535	0.25424	0.23799	0.044331	-0.12466	0.36682	0.25395
**NEFM**	0.72283	0.65319	0.69902	0.26086	0.63977	0.93698	1	0.95697	0.97632	0.93684	0.95398	-0.41142	-0.52094	0.33054	0.18066	0.055975	-0.21703	0.28617	0.15841
**GD1b**	0.8044	0.72928	0.61187	0.151	0.72882	0.88893	0.95697	1	0.97509	0.89615	0.90815	-0.45271	-0.49969	0.16201	0.037551	0.004126	-0.31544	0.31258	0.20092
**GM1**	0.71703	0.64225	0.61926	0.18898	0.62074	0.88565	0.97632	0.97509	1	0.96634	0.97645	-0.48726	-0.54525	0.25709	0.027429	-0.0271	-0.36563	0.17589	0.095968
**GT1b**	0.57741	0.4787	0.59412	0.27063	0.42354	0.79758	0.93684	0.89615	0.96634	1	0.99434	-0.46536	-0.5653	0.33352	-0.03376	-0.03694	-0.4176	-0.00953	-0.04891
**GD1a**	0.60955	0.50452	0.62144	0.28731	0.47833	0.84535	0.95398	0.90815	0.97645	0.99434	1	-0.48248	-0.57043	0.33823	-0.00512	-0.03942	-0.40728	0.015432	-0.02221
**CD44**	-0.52803	-0.57728	0.25347	0.099107	-0.1008	-0.40519	-0.41142	-0.45271	-0.48726	-0.46536	-0.48248	1	0.86903	0.55798	0.57461	0.86874	0.77143	0.26542	0.61974
**GFAP**	-0.64032	-0.58057	-0.02065	-0.17413	0.025711	-0.53535	-0.52094	-0.49969	-0.54525	-0.5653	-0.57043	0.86903	1	0.4319	0.4473	0.72285	0.58852	0.20645	0.5164
**S100B**	-0.22592	-0.28877	0.69972	0.31338	0.19377	0.25424	0.33054	0.16201	0.25709	0.33352	0.33823	0.55798	0.4319	1	0.67495	0.79213	0.44496	0.1553	0.43642
**GM2**	-0.07377	0.10087	0.50988	-0.08817	0.31314	0.23799	0.18066	0.037551	0.027429	-0.03376	-0.00512	0.57461	0.4473	0.67495	1	0.70006	0.88063	0.73652	0.70555
**GD3**	-0.17803	-0.28766	0.64194	0.25645	0.29109	0.044331	0.055975	0.004126	-0.0271	-0.03694	-0.03942	0.86874	0.72285	0.79213	0.70006	1	0.68949	0.43308	0.74929
**GD2**	-0.25086	-0.10313	0.27587	-0.12688	0.027783	-0.12466	-0.21703	-0.31544	-0.36563	-0.4176	-0.40728	0.77143	0.58852	0.44496	0.88063	0.68949	1	0.67639	0.70184
**MAP2**	0.40249	0.60257	0.36529	-0.29851	0.61006	0.36682	0.28617	0.31258	0.17589	-0.00953	0.015432	0.26542	0.20645	0.1553	0.73652	0.43308	0.67639	1	0.74398
**SOX2**	0.16624	0.17048	0.5813	-0.09372	0.5663	0.25395	0.15841	0.20092	0.095968	-0.04891	-0.02221	0.61974	0.5164	0.43642	0.70555	0.74929	0.70184	0.74398	1

P-value

	DCX	TUBB3	GM3	TTR	FABP7	NEFL	NEFM	GD1b	GM1	GT1b	GD1a	CD44	GFAP	S100B	GM2	GD3	GD2	MAP2	SOX2
**DCX**	NA	0.002835	0.20012	0.58214	0.058708	0.006804	0.027794	0.008948	0.029696	0.10351	0.08139	0.14395	0.063207	0.55889	0.85039	0.64676	0.51501	0.28283	0.66905
**TUBB3**	0.002835	NA	0.57348	0.51152	0.079292	0.035674	0.05644	0.025773	0.06216	0.19236	0.16602	0.10361	0.10119	0.45108	0.79625	0.45291	0.79177	0.085917	0.661
**GM3**	0.20012	0.57348	NA	0.104	0.12224	0.021342	0.036141	0.07992	0.075339	0.091603	0.074026	0.51048	0.95795	0.035875	0.16083	0.062328	0.47243	0.3337	0.10065
**TTR**	0.58214	0.51152	0.104	NA	0.9582	0.36704	0.4978	0.69818	0.62629	0.48122	0.45348	0.79975	0.65411	0.41155	0.82153	0.50536	0.74496	0.43524	0.81047
**FABP7**	0.058708	0.079292	0.12224	0.9582	NA	0.031988	0.063509	0.025913	0.074448	0.25597	0.19275	0.79639	0.94765	0.6174	0.41193	0.44729	0.94344	0.081066	0.11192
**NEFL**	0.006804	0.035674	0.021342	0.36704	0.031988	NA	0.000194	0.001347	0.001486	0.010017	0.004103	0.2793	0.13745	0.50916	0.53747	0.90984	0.74932	0.33153	0.50966
**NEFM**	0.027794	0.05644	0.036141	0.4978	0.063509	0.000194	NA	5.22E-05	6.57E-06	0.000196	6.58E-05	0.27126	0.15042	0.38497	0.64183	0.88627	0.57486	0.45536	0.68396
**GD1b**	0.008948	0.025773	0.07992	0.69818	0.025913	0.001347	5.22E-05	NA	7.84E-06	0.001072	0.000706	0.22109	0.17077	0.6771	0.92359	0.99159	0.40831	0.41282	0.60421
**GM1**	0.029696	0.06216	0.075339	0.62629	0.074448	0.001486	6.57E-06	7.84E-06	NA	2.23E-05	6.45E-06	0.18338	0.12895	0.50425	0.94416	0.94483	0.33321	0.65079	0.80599
**GT1b**	0.10351	0.19236	0.091603	0.48122	0.25597	0.010017	0.000196	0.001072	2.23E-05	NA	4.46E-08	0.20683	0.1127	0.38043	0.93128	0.92483	0.26341	0.9806	0.90056
**GD1a**	0.08139	0.16602	0.074026	0.45348	0.19275	0.004103	6.58E-05	0.000706	6.45E-06	4.46E-08	NA	0.18837	0.10874	0.37331	0.98957	0.9198	0.2766	0.96857	0.95477
**CD44**	0.14395	0.10361	0.51048	0.79975	0.79639	0.2793	0.27126	0.22109	0.18338	0.20683	0.18837	NA	0.00235	0.11847	0.10559	0.002367	0.014908	0.49004	0.075052
**GFAP**	0.063207	0.10119	0.95795	0.65411	0.94765	0.13745	0.15042	0.17077	0.12895	0.1127	0.10874	0.00235	NA	0.24569	0.22736	0.027788	0.095495	0.59407	0.15464
**S100B**	0.55889	0.45108	0.035875	0.41155	0.6174	0.50916	0.38497	0.6771	0.50425	0.38043	0.37331	0.11847	0.24569	NA	0.046083	0.010929	0.23009	0.68991	0.24023
**GM2**	0.85039	0.79625	0.16083	0.82153	0.41193	0.53747	0.64183	0.92359	0.94416	0.93128	0.98957	0.10559	0.22736	0.046083	NA	0.035748	0.001719	0.023622	0.033708
**GD3**	0.64676	0.45291	0.062328	0.50536	0.44729	0.90984	0.88627	0.99159	0.94483	0.92483	0.9198	0.002367	0.027788	0.010929	0.035748	NA	0.03989	0.24426	0.020125
**GD2**	0.51501	0.79177	0.47243	0.74496	0.94344	0.74932	0.57486	0.40831	0.33321	0.26341	0.2766	0.014908	0.095495	0.23009	0.001719	0.03989	NA	0.045443	0.035079
**MAP2**	0.28283	0.085917	0.3337	0.43524	0.081066	0.33153	0.45536	0.41282	0.65079	0.9806	0.96857	0.49004	0.59407	0.68991	0.023622	0.24426	0.045443	NA	0.021535
**SOX2**	0.66905	0.661	0.10065	0.81047	0.11192	0.50966	0.68396	0.60421	0.80599	0.90056	0.95477	0.075052	0.15464	0.24023	0.033708	0.020125	0.035079	0.021535	NA

Table S12. List of qPCR primers

Target gene	Forward (5′-3′ direction)	Reverse (3′-5′ direction)
SOX2	TACAGCATGTCCTACTCGCAG	GAGGAAGAGGTAACCACAGGG
DCX	TATGCGCCGAAGCAAGTCTCCA	CATCCAAGGACAGAGGCAGGTA
TUBB3	TCAGCGTCTACTACAACGAGGC	GCCTGAAGAGATGTCCAAAGGC
MAP2	TTGGTGCCGAGTGAGAAGA	GTCTGGCAGTGGTTGGTTAA
GFAP	CCGACAGCAGGTCCATGT	GTTGCTGGACGCCATTG
S100B	Qiagen QuantiTect Primer Assays Hs_S100B_1_SG; QT00059164	
TTR	CAGAGCTGCATGGGCTCACA	GATTGGTGACGACAGCCGTG
GAPDH	AGCCACATCGCTCAGACAC	GCCCAATACGACCAAATCC

Table S13. Proteomics protocol reproducibility

Protein [gene name]	Quantifier ST peptide	CV [%]
SOX2	LLSETEK	6
FABP7	ALGVGFATR	11
DCX	YIYTIDGSR	14
TUBB3	ISVYYNEASSHK	14
MAP2	LINQPLPDLK	9
NEFL	FTVLTESAAK	12
NEFM	SIELESVR	21
S100B	AMVALIDVFHQYSGR	12
GFAP	DNLAQDLATVR	5
CD44	ALSIGFETCR	17
TTR	AADDTWEPFASGK	8
ACTB	AVFPSIVGR	11
GAPDH	LISWYDNEFGYSNR	3
**AVERAGE [%]**		**11**
